# Why Prioritize Personal Gain Over Principles? The Relationship Between Calculative Mindset, Moral Disengagement, and Ethical Compromise

**DOI:** 10.1002/pchj.70120

**Published:** 2026-09-09

**Authors:** Shiqi Wang, Xiaoling Sun, Kexin Hu, Mingzheng Wu

**Affiliations:** ^1^ Department of Psychology and Behavioral Sciences Zhejiang University Hangzhou China; ^2^ Department of Psychology Hangzhou Normal University Hangzhou China; ^3^ School of Management and E‐Business, Zhejiang Gongshang University Hangzhou Zhejiang China

**Keywords:** calculative mindset, ethical compromise, moral disengagement

## Abstract

People often need to weigh the trade‐off between obtaining economic/status benefits and complying with moral standards in their daily lives. Once individuals make ethical compromises, they will abandon moral principles to obtain economic/status benefits. Therefore, it is necessary to explore the risk factors and mechanisms that induce ethical compromise. Using questionnaires (Study 1, Study 2), we found that calculative mindset is positively associated with individuals' moral disengagement, which corresponded with a tendency to make economic/status ethical compromises. Using experimental methods (Study 3), we found that calculative mindset increases individuals' moral disengagement, leading to a willingness to accept immoral help from others. This indicates that reducing people's calculative mindset will help reduce ethical compromises.

## 1 Introduction

Calculative mindset refers to an individual's unconscious tendency to analyze nonquantitative problems mathematically (Wang et al. 2014; Kim et al. 2022). The theoretical relevance of calculative mindset can be further clarified by Relational Models Theory (Fiske 1992), which proposes that people organize social life through four elementary relational models: communal sharing, authority ranking, equality matching, and market pricing. Among these models, market pricing is particularly relevant to calculative mindset because it organizes social relationships according to ratios, rates, proportionality, and cost–benefit calculations. In market‐pricing relationships, people reduce qualitatively different inputs and outcomes to a common value metric, thereby enabling comparisons across diverse objects, actions, and social exchanges (Fiske 1992). Thus, market pricing provides an important relational foundation for understanding why a calculative orientation may extend beyond explicitly economic domains and enter interpersonal or moral domains.

Relatedly, recent research on market relationships and market mindset suggests that when people adopt a market‐oriented way of thinking, they tend to construe social interactions in terms of exchange, rationality, efficiency, self‐interest, and proportional return. Market relationships are typically characterized by clear rules concerning inputs, outputs, timing, and expected repayment, and they are perceived as more structured and controllable than communal relationships (Gasiorowska and Zaleskiewicz 2021). In this sense, market mindset is closely related to calculative mindset, because both involve applying economic or quasi‐economic logic to social situations. However, the two constructs are not identical. Market mindset refers primarily to a relational frame or social schema activated by market‐pricing contexts, in which individuals interpret interactions as exchanges governed by proportionality and comparable return. By contrast, calculative mindset reflects a general tendency to translate non‐quantitative issues into calculable terms and to evaluate choices through mathematical, cost–benefit, or utility‐based reasoning, even when the situation is not explicitly framed as a market exchange.

Proportional thinking is also conceptually related to, but distinct from, calculative mindset. Proportional thinking refers to sensitivity to ratios, rates, and comparative proportions, and is considered a key cognitive feature of market‐pricing relationships and market mindset (Fiske 1992; Kuzminska et al. 2024). For example, people with an activated market mindset are more likely to attend to the ratio between investment and return, costs and benefits, or effort and reward (Kuzminska et al. 2024). Accordingly, proportional thinking can be regarded as a more specific cognitive process through which market mindset operates. In contrast, calculative mindset is not limited to proportional comparison. It includes a broader tendency to mathematize social, moral, and interpersonal issues by considering costs, benefits, gains, losses, efficiency, and utility.

According to rational choice theory (Rutar 2019), individuals with a high calculative mindset may conduct cost–benefit analyses of things around them and seek benefit‐maximizing choices through calculation. They not only calculate economic and financial matters to maximize profits, but also extend their calculations to non‐quantitative social relationships and values (Wang et al. 2014). For instance, an individual with a high calculative mindset might choose friends or a spouse in terms of the potential costs and benefits of establishing relationships. Similarly, a construction site manager with a high calculative mindset may prioritize the cost‐effectiveness of safety measures for workers (Kim et al. 2022). From the perspective of Relational Models Theory, such examples indicate that the calculative mindset may lead individuals to apply market‐pricing logic to domains that are often expected to be regulated by communal or moral concerns, such as friendship, care, and responsibility.

This extension of calculation into moral and interpersonal domains may have important consequences for ethical compromise. When individuals construe relationships and moral decisions in terms of utility, efficiency, and proportional return, they may become more likely to give greater weight to instrumental benefits than to ethical ideals or relational obligations. For example, once individuals perceive that their friends no longer contribute sufficient value, they may choose to end the relationship. Similarly, in organizational settings, managers may prioritize cost efficiency, performance targets, or status‐related gains over employee welfare, thereby making decisions that involve ethical compromises. Although ethical compromise has been discussed in political science and philosophy, existing work has mainly examined this phenomenon through qualitative or normative approaches (Chan and Protzen 2018; Simkulet 2020). Less attention has been paid to how individual differences and social‐contextual factors shape people's willingness to make ethical compromises. Therefore, the present research investigates the effect of calculative mindset on ethical compromise and its underlying psychological mechanisms from both dispositional and situational perspectives.

Ethical compromise refers to the behavioral tendency to place moral values (such as honesty and fairness) below secular values (such as money and status) (Kennedy and Kray 2014). Importantly, ethical compromise should not be equated with unethical behavior or moral violation. Although some ethical compromises may involve clear violations of moral norms, others may reflect value trade‐offs, pragmatic concessions, or a reduced prioritization of ethical ideals under competing demands. For example, when a professional recommends additional services partly because doing so benefits their own financial or status interests, this may reflect a willingness to place fairness or care below instrumental benefits. Such a decision can be perceived as ethically compromising even when it does not necessarily constitute an explicit moral violation. Thus, in the present research, ethical compromise refers to people's willingness to make decisions in which ethical concerns are subordinated to economic or status‐related considerations, rather than to objectively unethical behavior per se.

Ethical compromise is a highly important concept, and discussions on this concept involve multiple fields, such as philosophical ethics (Archard 2012), policymaking (Hall 2022), planning (Chan and Protzen 2018), social relationships (May 2011), organizational management (Kennedy and Kray 2014), and so on. In people's daily lives, economic and status ethical compromises are relatively common (Kennedy and Kray 2014). A calculative mindset leads people to quantify social values that are not easily quantified, thereby reducing the influence of intrinsic and moral values (Bennis et al. 2010). As a result, individuals may become more likely to prioritize money, status, or other instrumental benefits over ethical concerns. The Affective Harm Account of moral judgment suggests that people's moral judgment is based on the perception of harm and the emotional evaluation of behavior (Gray et al. 2022). Research has found that a calculative mindset can lead people to be self‐interested (Kim et al. 2022), making people less empathetic toward others (Svedholm‐Häkkinen and Lindeman 2017), and pay less attention to the harm their actions cause to others (Caruso et al. 2013). These findings imply that a calculative mindset may weaken the influence of moral standards and harm‐related concerns on decision‐making, making individuals more willing to prioritize economic or status‐related benefits in situations involving ethical compromises. Therefore, we formulated the following hypothesis:

Hypothesis 1 *Higher levels of calculative mindset are associated with higher levels of ethical compromise*.

Calculative mindset may lead individuals to make ethical compromises by increasing their tendency toward moral disengagement. Bandura's (1991) social cognitive theory suggests an individual's self‐regulative system constrains the individual with moral standards before engaging in unethical behavior. After engaging in unethical behavior, the self‐regulative system prompts the individual to undertake ethical behavior for remediation, or to constrain the subsequent unethical behavior, through emotions such as guilt and shame. More specifically, moral agency is regulated through self‐monitoring, moral judgment, and self‐reactive processes; individuals usually refrain from violating their moral standards because such behavior can elicit self‐censure and negative self‐evaluative reactions (Bandura 1991; Bandura et al. 1996). Once the self‐regulative system is disrupted, moral disengagement occurs, leading to unethical behavior (Tillman et al. 2018; Yang and Liang 2022). Moral disengagement allows individuals to selectively disengage moral self‐sanctions from morally questionable conduct by cognitively reconstruing the conduct, obscuring personal responsibility, minimizing or distorting harmful consequences, or devaluing the victims of their actions (Bandura et al. 1996; Moore 2015).

The quantitative calculation of emotional and moral values by individuals with a high calculative mindset reduces the restriction of moral norms on behavior (Bennis et al. 2010) and limits the self‐monitoring function and self‐reactive influence of self‐regulative systems. Specifically, calculative mindset refers to the tendency to analyze and convert non‐quantifiable social or moral values into numeric or monetary metrics (Wang et al. 2014; Kim et al. 2022). Individuals with a high calculative mindset tend to evaluate social relationships, moral values, and behavioral choices in terms of costs, benefits, and utility (Wang et al. 2014; Kim et al. 2022). Therefore, when facing ethically relevant situations, they may shift their attention from moral concerns, such as harm, responsibility, fairness, and interpersonal obligations, to instrumental concerns, such as efficiency, personal benefits, and outcome maximization.

This calculative framing may activate several specific mechanisms of moral disengagement. First, it may facilitate moral justification. When individuals construe ethical compromise as a way to maximize benefits, reduce losses, or produce more efficient outcomes, morally questionable behavior can be reinterpreted as rational, necessary, or even beneficial. This is consistent with moral justification, in which harmful conduct is made personally or socially acceptable by linking it to valued or worthy purposes (Bandura et al. 1996). Second, calculative mindset may promote advantageous comparison. Because calculative thinking encourages individuals to compare alternatives according to their relative utility, individuals may compare their own ethical compromise with more serious moral violations and conclude that their behavior is relatively minor or acceptable. Bandura et al. (1996) suggested that injurious conduct can appear benign or less consequential when it is contrasted with more reprehensible behavior. Third, calculative mindset may encourage euphemistic labeling. Morally questionable behavior may be described in neutral or instrumental terms, such as cost–benefit analysis, efficiency improvement, rational choice, or strategic adjustment, which makes ethical compromise appear less morally problematic and more consistent with pragmatic decision‐making. Moreover, calculative mindset may facilitate the minimization or distortion of consequences. Because harm to others, emotional responses, moral obligations, and interpersonal consequences are often difficult to quantify, individuals with a high calculative mindset may pay less attention to these nonquantifiable consequences and focus more on measurable outcomes. This process is consistent with prior evidence that calculative thinking can reduce attention to social and moral concerns, increase self‐interested or deceptive behavior, and dampen emotional reactions to selfish decisions (Wang et al. 2014).

Calculative mindset may weaken moral self‐regulation by changing how individuals construe morally relevant situations. By transforming moral issues into cost–benefit problems, calculative mindset may make it easier for individuals to justify ethical compromise, compare it favorably with more serious wrongdoing, label it in pragmatic terms, and minimize its harmful consequences. In this way, calculative mindset may increase moral disengagement and thereby make individuals more willing to make ethical compromises. Numerous studies have shown that moral disengagement can effectively predict unethical behavior (Yan et al. 2021; Guo et al. 2021). Ethical compromise is an act where individuals compromise moral values in pursuit of secular values, which is a behavior with low moral evaluations. Accordingly, we posited the following:

Hypothesis 2 *Moral disengagement mediates the association between calculative mindset and ethical compromise. Specifically, a stronger calculative mindset is expected to be associated with higher moral disengagement, which is further expected to be related to* a *greater willingness to make ethical compromises*.

In sum, this research consists of three studies. Study 1 employs questionnaires to explore the relationship between calculative mindset and economic ethical compromise, as well as the mediating role of dispositional moral disengagement. Study 2, using questionnaires as well, investigates the relationship between calculative mindset and status ethical compromise, along with the mediating role of situational moral disengagement. Study 3, using an experimental method, investigates the causal relationship between calculative mindset and acceptance of immoral help, as well as the mediating role of situational moral disengagement. Accepting immoral help involves benefiting oneself while indirectly causing harm to others, and aligns with the definition of ethical compromise. Therefore, we think that this scenario can be used to measure ethical compromise.

## 2 Study 1

The aim of Study 1 is to explore the relationship between calculative mindset and economic ethical compromises and the mediating role of moral disengagement.

### 2.1 Method

#### 2.1.1 Participants

We used G*Power (version 3.1) to calculate the required sample size (Faul et al. 2007) with a medium effect size (*r* = 0.30), a statistical power of 0.95, and *α* = 0.05 (Cohen 1988). This analysis suggested a sample size of 111. We recruited 146 participants from the Credamo platform (https://www.credamo.com). The Credamo platform is a Chinese research‐focused platform with extensive industry coverage and over 3 million registered users. This platform is similar to other data collection platforms like Prolific, Qualtrics, and Amazon Mechanical Turk. Among these participants, 61 were male (41.8%), 85 were female (58.2%); 79 were below 30 years old (54.1%), 56 were between 31–40 years old (38.4%), and 11 were above 40 years old (7.5%); 14 participants had an educational level below a bachelor's degree (9.6%), 106 participants had a bachelor's degree (72.6%), and 26 participants had a postgraduate degree or above (17.8%).

#### 2.1.2 Materials and Measures

*Calculative mindset* was measured using the calculative mindset scale. The study revised the calculative mindset scale developed by Kim et al. (2022). The calculative mindset scale is a single‐factor scale that measures an individual's calculative mindset with 10 items, using a 7‐point Likert scale. We used the average of the 10 items to reflect the level of individuals' calculative mindset, with higher scores indicating higher levels of calculative mindset (Kim et al. 2022).

We translated the calculative mindset scale and tested the reliability and validity of the scale. The process of scale revision is as follows. First, the scale was translated into Chinese using a standardized translation‐back‐translation procedure (Brislin 1980). Secondly, 285 participants (83 males) who did not participate in this study were selected to participate in the scale revision process. Exploratory factor analysis revealed a Kaiser–Meyer–Olkin measure of sampling adequacy (KMO) of 0.95, Bartlett's test of sphericity = 2435.46, *p* < 0.001, extracting a single factor with standardized factor loadings greater than 0.74 for each item, explaining 69.72% of the total variance. The Cronbach's α coefficient was 0.95. We also tested the relationship between calculative mindset and social connectedness, loneliness and depression. We used social connectedness scale‐revised (Fan et al. 2015) to measure social connectedness, UCLA loneliness scale (Russell et al. 1980) to measure loneliness and self‐rating depression scale (Zung 1965) to measure depression. Calculative mindset was significantly negatively correlated with social connectedness (*r* = −0.47, *p* < 0.001) and positively correlated with loneliness (*r* = 0.49, *p* < 0.001) and depression (*r* = 0.35, *p* < 0.001). This indicates that the revised scale has good reliability and validity. The scale consists of 10 items, with a sample item being “I tend to calculate the value of my relationships with others.” Scores were given on a 7‐point Likert scale, ranging from 1 “*completely disagree*” to 7 “*completely agree*.”

*Economic ethical compromise* was measured using a scenario and two questions. The economic ethical compromise scenario was developed by Kennedy and Kray (2014). In this scenario, the protagonist makes decisions that compromise ethical values for social status or monetary gains, making an ethical compromise. The scenario involves the protagonist, A, who establishes a small venture capital company operating globally. B, previously an assistant in a financial firm, is hired by A for the new company and diligently supports the work of the company's four partners while handling numerous administrative tasks related to the company's establishment and operation. B often works overtime on weekends or responds to travel arrangement emails late at night but receives the same salary as in the previous financial company. Recently, A discovered that local assistants were paid much less than B. Considering that the company is already established and operating with a reduced workload for the assistant, A decides to terminate B and hire a less expensive assistant.

The questions to measure economic ethical compromise were as follows. After reading the scenario, participants were asked to judge the acceptability of the protagonist's behavior (“Protagonist A's behavior is acceptable”) and the impression of the protagonist (“Protagonist A is a morally good person”). A 7‐point Likert scale was used, with 1 representing “*completely disagree*” and 7 representing “*completely agree*.” We treated these two items as separate outcome variables. Behavioral acceptability reflected participants' evaluation of whether the ethically compromising behavior was acceptable, whereas positive moral character evaluation captured the extent to which participants viewed the protagonist as morally good. Higher scores indicated greater perceived behavioral acceptability and a more positive evaluation of the protagonist's moral character, respectively.

*Moral disengagement* was measured using the moral disengagement scale developed by Shu et al. (2011). The scale contains six items scored on a 7‐point Likert scale ranging from 1 (*completely disagree*) to 7 (*completely agree*). A higher average score indicates a higher level of moral disengagement. The Cronbach's *α* coefficient in this study was 0.84.

In the study, demographic information such as sex, age, and educational level of the participants was collected. Previous research has shown that men are more likely than women to make ethical compromises (Kennedy and Kray 2014) and view morally questionable behaviors as more permissible (Ward and King 2018). Therefore, sex was used as a control variable in this study. Previous studies have indicated that compared to younger individuals, the elderly are less likely to make utilitarian moral judgments; thus, age was included as a control variable (Zhao 2021).

#### 2.1.3 Procedure

At the beginning of the study, all participants first read the informed consent form and were asked to provide consent for participation and data usage. Then, participants filled out the survey questionnaire. First, they completed the calculative mindset scale, then completed the moral disengagement scale. Next, they read the economic ethical compromise scenarios and completed the measurement of ethical compromise. Finally, the demographic information of the participants was collected.

### 2.2 Results and Discussion

Descriptive analysis and correlation analysis results (Table 1) showed significant positive correlations between calculative mindset and moral disengagement (*r* = 0.63, *p* < 0.001), calculative mindset and behavioral acceptability (*r* = 0.35, *p* < 0.001) and calculative mindset and positive moral character evaluation (*r* = 0.24, *p* < 0.01). Furthermore, moral disengagement was significantly positively correlated with behavioral acceptability (*r* = 0.36, *p* < 0.001) and positive moral character evaluation (*r* = 0.25, *p* < 0.01). Additionally, behavioral acceptability showed a significant negative correlation with age (*r* = −0.19, *p* < 0.05).

**TABLE 1:** Descriptive analysis and correlation analysis of the main variables in Study 1 (*n* = 146).

	*M*	SD	1	2	3	4	5	6
1. Sex	1.58	0.49	—					
2. Age	2.51	0.82	−0.17*	—				
3. Educational Level	2.08	0.52	0.08	0.08	—			
4. Calculative Mindset	3.51	1.57	−0.07	−0.10	0.01	—		
5. Moral Disengagement	3.58	1.27	−0.06	−0.10	−0.08	0.63**	—	
6. Behavioral Acceptability	2.43	1.53	−0.02	−0.19*	−0.15	0.35**	0.36**	
7. Positive Moral Character Evaluation	2.23	1.14	−0.03	−0.15	−0.09	0.24**	0.25**	0.57**

*Note:* *p < 0.05; **p < 0.01. Categorical variable: Sex: 1 = “male”, 2 = “female”; Age: 1 = “0–20 years”, 2 = “21–30 years”, 3 = “31–40 years”, 4 = “41–50 years”, 5 = “51–60 years”, 6 = “Above 60 years”; Educational level: 1 = “Below the bachelor degree”, 2 = “Bachelor”, 3 = “Master degree or above”.

**FIGURE 1:**
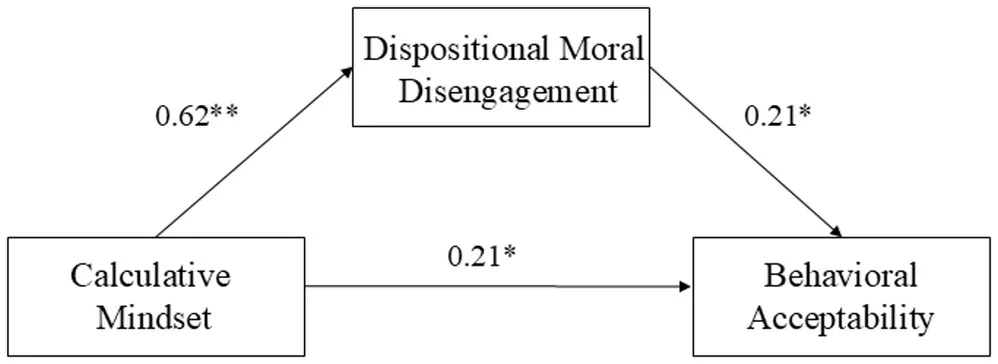
The mediation model of moral disengagement in the relationship between calculative mindset and behavioral acceptability. *Note:* All coefficients are standardized coefficients. *p < 0.05; **p < 0.01.

We conducted a mediation analysis (PROCESS v4.1 for SPSS, 5000 bootstraps) with calculative mindset as the independent variable, moral disengagement as the mediator, and behavioral acceptability as the dependent variable, controlling for sex, age, and educational level. The total effects of calculative mindset on behavioral acceptability (*b* = 0.33, 95% CI [0.18, 0.48], SE = 0.08, *p* < 0.001, b* = 0.34) were significant. Participants higher in calculative mindset reported higher levels of moral disengagement (*b* = 0.50, 95% CI [0.40, 0.61], SE = 0.05, *p* < 0.001, b* = 0.62). Moral disengagement was positively associated with behavioral acceptability (*b* = 0.25, 95% CI [0.02, 0.49], SE = 0.12, *p* < 0.05, b* = 0.21). The indirect effect via moral disengagement was 0.13 (b* = 0.13), with a 95% CI [0.03, 0.24] that did not include zero, indicating a significant indirect effect (Figure 1).

**FIGURE 2:**
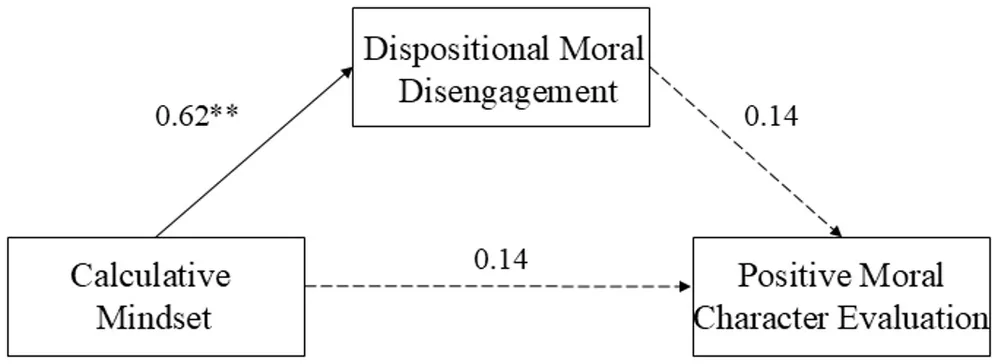
The mediation model of moral disengagement in the relationship between calculative mindset and positive moral character evaluation.

We conducted a mediation analysis (PROCESS v4.1 for SPSS, 5000 bootstraps) with calculative mindset as the independent variable, moral disengagement as the mediator and positive moral character evaluation as the dependent variable, controlling sex, age, and educational level. The total effects of calculative mindset on positive moral character evaluation (*b* = 0.16, 95% CI [0.05, 0.28], SE = 0.06, *p* < 0.01, b* = 0.23) were significant. Participants higher in calculative mindset reported higher levels of moral disengagement (*b* = 0.50, 95% CI [0.40, 0.61], SE = 0.05, *p* < 0.001, b* = 0.62). The effect of moral disengagement on positive moral character evaluation was not significant (*b* = 0.13, 95% CI [−0.06, 0.31], SE = 0.09, *p* = 0.18, b* = 0.14). The indirect effect via moral disengagement was 0.06 (b* = 0.09), with a 95% CI [−0.02, 0.16] that included zero, indicating indirect effect was not significant (Figure 2).

To address alternative causal possibilities, we also tested competing mediation models involving calculative mindset, moral disengagement, behavioral acceptability, and positive moral character evaluation. Specifically, we first tested models in which calculative mindset served as the mediator between moral disengagement and each outcome variable. When behavioral acceptability was used as the dependent variable, the indirect effect of moral disengagement through calculative mindset was significant, *b* = 0.16, b* = 0.13, 95% CI [0.02, 0.30]. However, when positive moral character evaluation was used as the dependent variable, the indirect effect of moral disengagement through calculative mindset was not significant, *b* = 0.08, b* = 0.08, 95% CI [−0.04, 0.21].

We then tested models in which behavioral acceptability and positive moral character evaluation served as mediators between calculative mindset and moral disengagement. The indirect effect of calculative mindset on moral disengagement through behavioral acceptability was significant, *b* = 0.04, b* = 0.05, 95% CI [0.01, 0.08]. In contrast, the indirect effect through positive moral character evaluation was not significant, *b* = 0.02, b* = 0.02, 95% CI [−0.01, 0.04].

These results suggest that the alternative mediation models were only partially supported and were mainly evident when behavioral acceptability was involved. This pattern further indicates that behavioral acceptability and positive moral character evaluation should not be treated as interchangeable indicators of a single ethical compromise outcome. More importantly, although the hypothesized mediation model was supported, the cross‐sectional data do not allow us to definitively determine the causal ordering among calculative mindset, moral disengagement, and the two outcome variables. Therefore, the mediation results should be interpreted as indirect associations consistent with our theoretical model, rather than as definitive evidence of a causal process. Detailed analyses of the alternative mechanisms can be found in the Supporting Information.

Study 1 found that calculative mindset was significantly positively related to ethical compromise, and moral disengagement played a mediating role. In Study 1, we measured participants' economic ethical compromise. However, besides money, social status is also a scarce currency. Individuals may likely compromise their moral values in pursuit of organizational status, engaging in status ethical compromise. Accordingly, Study 2 extended Study 1 by examining status‐related ethical compromise. Importantly, in line with the different patterns of results observed for the two variables in Study 1, status ethical compromise was operationalized using two distinct outcome variables—behavioral acceptability and positive moral character evaluation—to capture both behavioral judgments and moral trait inferences.

In terms of measuring moral disengagement, Study 1 measured individuals' trait moral disengagement but did not measure the moral disengagement exhibited by individuals in specific situations. Therefore, we developed measurement items for situational moral disengagement to investigate the relationships between calculative mindset, situational moral disengagement, and ethical compromise.

## 3 Study 2

### 3.1 Method

#### 3.1.1 Participants

We used G*Power (version 3.1) to calculate the required sample size (Faul et al. 2007) with a medium effect size (*r* = 0.30), a statistical power of 0.95, and *α* = 0.05 (Cohen 1988). This analysis suggested a sample size of 111. We recruited 140 participants from the Credamo platform (https://www.credamo.com). Among these participants, 59 were male (42.1%), 81 were female (57.9%); 76 participants were below 30 years old (54.3%), 61 were aged between 31 and 40 (43.6%), and only 3 were above 40 years old (2.1%); 12 participants had an educational level below a bachelor's degree (8.6%), 110 participants had a bachelor's degree (78.6%), and 18 participants had a postgraduate degree or above (12.8%).

#### 3.1.2 Materials and Measures

*Calculative mindset* was measured using the same scale from Study 1. The Cronbach's *α* coefficient in this study was 0.96.

*Status ethical compromise* was measured using a scenario and two questions. The status ethical compromise scenario was developed by Kennedy and Kray (2014). In this scenario, the protagonist chooses work status over moral norms, making an ethical compromise. The scenario involves protagonist A, a new employee in a private equity fund. A wants to advance in the company and decides to ally with an influential partner. In the following investment committee meetings, A supports whatever the partner says, even if it's a crazy idea to invest in an electric car company that would likely cause substantial losses for other partners. No one in the company supports this idea, and A also thinks it's a bad investment proposal. However, A believes that at this moment, agreeing with the partner's opinion is the right thing to do. So, A intervenes and says, “This is a great idea! We will be at the forefront, and potential investors will find us more interesting. It's an innovative idea. I'll call the company today to see if they are interested.”

The questions to measure status ethical compromise were the same as in Study 1. Participants evaluated the acceptability of the protagonist's behavior and the extent to which they viewed the protagonist as morally good. Although the two items were significantly correlated (*r* = 0.62, *p* < 0.001), they were treated as separate outcome variables because they capture conceptually distinct aspects of ethical judgment. Higher scores indicated greater perceived behavioral acceptability and a more positive moral character evaluation, respectively.

We developed questions of *situational moral disengagement* to measure moral disengagement. Following the situational moral disengagement measurement by Kish‐Gephart et al. (2014), we selected two dimensions from the eight dimensions of moral disengagement mechanisms that best fit the scenario and designed two questions based on them. According to Kish‐Gephart's research (2014), specific moral situations will trigger specific moral disengagement mechanisms. Therefore, we selected two moral disengagement mechanisms that are more likely to occur in the scenario in this study and set the following questions. Attribution of blame: “1. Protagonist A's actions are fine; the problem lies with the partner, who did not consider the reasonableness of the suggestion when making the proposal.”; moral justification: “2. Protagonist A's actions are fine; the decision to invest or not should be collectively decided by the investment committee, and Protagonist A just made a suggestion.” A 7‐point Likert scale was used to measure participants' agreement with the statements, with 1 representing “*completely disagree*” and 7 representing “*completely agree*.” The two questions showed a significant positive correlation (*r* = 0.62, *p* < 0.001). Thus, we added the scores of the two questions together as the situational moral disengagement score, where a higher score indicated a greater tendency toward situational moral disengagement.

We selected demographic variables such as sex, age, educational level as control variables, which are same as Study 1.

#### 3.1.3 Procedure

Participants first completed the calculative mindset scale, then they read the status ethical compromise scenarios and completed the measurement of ethical compromise and situational moral disengagement. Finally, the demographic information of the participants was measured.

### 3.2 Results and Discussion

Descriptive analysis and correlation analysis results (Table 2) showed significant positive correlations between the calculative mindset and moral disengagement (*r* = 0.34, *p* < 0.001), the calculative mindset and behavioral acceptability (*r* = 0.25, *p* < 0.01), and the calculative mindset and positive moral character evaluation (*r* = 0.19, *p* < 0.05). Additionally, moral disengagement showed a significant positive correlation with behavioral acceptability (*r* = 0.68, *p* < 0.001) and positive moral character evaluation (*r* = 0.63, *p* < 0.001). Furthermore, there was a significant negative correlation between the calculative mindset and age (*r* = −0.24, *p* < 0.01).

**TABLE 2:** Descriptive analysis and correlation analysis of the main variables in Study 2 (*n* = 140).

	*M*	SD	1	2	3	4	5	6
1. Sex	1.58	0.50	—					
2. Age	2.41	0.68	−0.18**	—				
3. Educational level	2.04	0.47	−0.06	0.04	—			
4. Calculative mindset	3.60	1.58	0.03	−0.24**	−0.06	—		
5. Moral disengagement	3.04	1.38	0.03	−0.17*	−0.07	0.34**	—	
6. Behavioral acceptability	2.58	1.45	−0.01	−0.01	−0.07	0.25**	0.68**	
7. Positive moral character evaluation	2.41	1.16	−0.02	−0.07	−0.04	0.19*	0.63**	0.66**

*Note:* *p < 0.05; **p < 0.01.

We assessed multicollinearity by calculating variance inflation factors (VIFs) using the car package in R for the regression models predicting behavioral acceptability and positive moral character evaluation, respectively. In both models, calculative mindset, moral disengagement, gender, age, and education were included as predictors. Because some control variables were categorical, generalized variance inflation factors (GVIFs) were obtained, and we interpreted the adjusted values, GVIF^(1/(2 × Df))^. The adjusted VIF values were identical across the two outcome models and ranged from 1.021 to 1.111, well below the commonly used cutoff of 5 and the more conservative cutoff of 3. These results suggest that multicollinearity was not a serious concern. Detailed results are reported in the Supporting Information.

**FIGURE 3:**
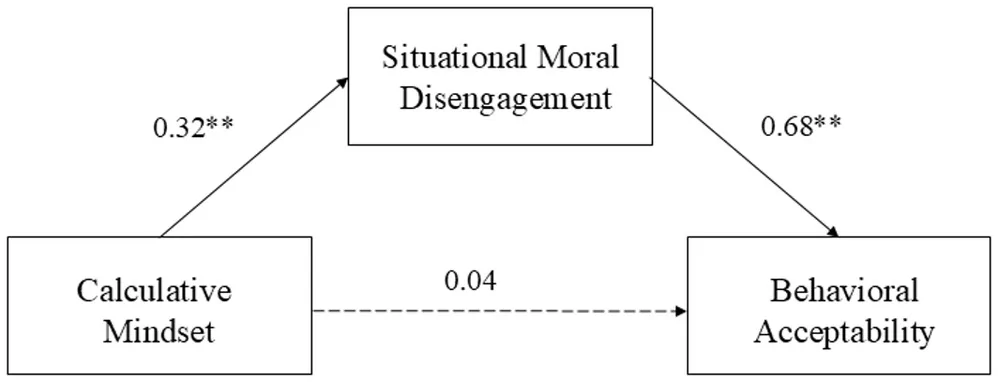
The mediation model of moral disengagement in the relationship between calculative mindset and behavioral acceptability.

We conducted a mediation analysis (PROCESS v4.1 for SPSS, 5000 bootstraps) with calculative mindset as the independent variable, moral disengagement as the mediator and behavioral acceptability as the dependent variable, controlling for sex, age, and educational level. The total effects of calculative mindset on behavioral acceptability (*b* = 0.24, 95% CI [0.08, 0.39], SE = 0.08, *p* < 0.01, b* = 0.26) were significant. Participants higher in calculative mindset reported higher levels of situational moral disengagement (*b* = 0.27, 95% CI [0.13, 0.42], SE = 0.05, *p* < 0.001, b* = 0.32). Situational moral disengagement was positively associated with behavioral acceptability (*b* = 0.72, 95% CI [0.58, 0.86], SE = 0.07, *p* < 0.001, b* = 0.68). The indirect effect through situational moral disengagement was 0.20 (b* = 0.22), with a 95% confidence interval of [0.09, 0.32] that did not include zero, indicating a significant indirect effect (Figure 3).

**FIGURE 4:**
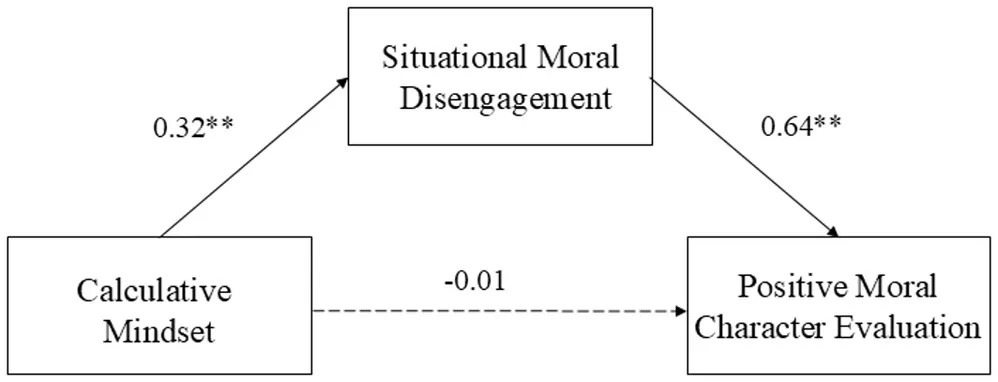
The mediation model of moral disengagement in the relationship between calculative mindset and positive moral character evaluation.

We conducted a mediation analysis (PROCESS v4.1 for SPSS, 5000 bootstraps) with calculative mindset as the independent variable, moral disengagement as the mediator and positive moral character evaluation as the dependent variable, controlling sex, age, and educational level. The total effects of calculative mindset on positive moral character evaluation (*b* = 0.14, 95% CI [0.01, 0.26], SE = 0.06, *p* < 0.05, b* = 0.18) were significant. Participants higher in calculative mindset reported higher levels of situational moral disengagement (*b* = 0.27, 95% CI [0.13, 0.42], SE = 0.05, *p* < 0.001, b* = 0.32). Situational moral disengagement was positively associated with positive moral character evaluation (*b* = 0.54, 95% CI [0.42, 0.66], SE = 0.06, *p* < 0.001, b* = 0.64). The indirect effect through situational moral disengagement was 0.15 (b* = 0.20), with a 95% confidence interval of [0.06, 0.25] that did not include zero, indicating a significant indirect effect (Figure 4).

To address alternative causal possibilities, we also tested competing mediation models involving calculative mindset, moral disengagement, behavioral acceptability, and positive moral character evaluation. Specifically, we first examined models in which calculative mindset served as the mediator between moral disengagement and each outcome variable. The indirect effect through calculative mindset was not significant when behavioral acceptability was used as the dependent variable, *b* = 0.01, b* = 0.01, 95% CI [−0.03, 0.06], or when positive moral character evaluation was used as the dependent variable, *b* = −0.005, b* = −0.01, 95% CI [−0.05, 0.03].

We then tested models in which behavioral acceptability and positive moral character evaluation served as mediators between calculative mindset and moral disengagement. The indirect effect of calculative mindset on moral disengagement through behavioral acceptability was significant, *b* = 0.14, b* = 0.16, 95% CI [0.04, 0.24]. Similarly, the indirect effect through positive moral character evaluation was significant, *b* = 0.09, b* = 0.11, 95% CI [0.003, 0.19].

These results indicate that the alternative mediation models were partially supported. Specifically, the models in which behavioral acceptability and positive moral character evaluation served as mediators were significant, whereas the models in which calculative mindset served as the mediator were not. This pattern suggests that, although the hypothesized mediation model was supported, the cross‐sectional data do not allow us to definitively determine the causal ordering among calculative mindset, moral disengagement, and the two outcome variables. Therefore, the mediation results should be interpreted as indirect associations consistent with our theoretical model, rather than as definitive evidence of a causal process. Detailed analyses of the alternative mechanisms can be found in the Supporting Information.

Study 2 generally supported and extended the findings of Study 1 in the context of status‐related ethical compromise. Specifically, calculative mindset was positively associated with both behavioral acceptability and positive moral character evaluation, and situational moral disengagement mediated these relationships. These findings suggest that individuals with a stronger calculative mindset were more likely to morally disengage in status‐related situations, which in turn was associated with greater acceptance of ethically compromising behavior and more positive evaluations of the protagonist's moral character.

Study 1 and Study 2 utilized questionnaire‐based methods, making it difficult to draw causal inferences regarding the relationships among the variables. In addition, although calculative mindset was measured through participants' first‐person self‐reports, the outcome variables in these two studies were assessed through participants' evaluations of another person's behavior and moral character in hypothetical scenarios. This difference in referent may introduce a potential perspective mismatch, as judgments of others' behavior and character may not fully capture how participants themselves would think or act when placed in the same situation. Moreover, Study 1 and Study 2 examined economic and status‐related ethical compromise in commercial organizational contexts. To address these limitations, Study 3 adopted an experimental design to examine the causal effect of calculative mindset on ethical compromise and used a more self‐relevant daily‐life scenario involving the acceptance of immoral help. Accepting immoral help may bring material benefits to oneself while also causing harm to others and can therefore be regarded as an indirect form of ethical compromise. In addition, given that a calculative mindset may reduce the influence of emotion, and prior research has shown that emotions play an important role in moral judgment (Baron et al. 2018; Li et al. 2021), Study 3 also included emotions as control variables.

## 4 Study 3

### 4.1 Method

#### 4.1.1 Participants

We used G*Power (version 3.1) to calculate the required sample size (Faul et al. 2007) with a medium effect size (*r* = 0.30), a statistical power of 0.95, and *α* = 0.05 (Cohen 1988). This analysis suggested a sample size of 122 per condition. We recruited 298 participants from the Credamo platform (https://www.credamo.com). Among them, 122 were male (40.9%) and 176 were female (59.1%); 168 were below 30 years old (56.4%), 110 were between 31–40 years old (36.9%), and 20 were above 40 years old (6.7%); 29 participants had an educational level below a bachelor's degree (9.7%), 228 participants had a bachelor's degree (76.5%), and 41 participants had a postgraduate degree or above (13.8%).

#### 4.1.2 Materials and Measures

*Calculative Mindset Manipulation* was as follows. Study 3 manipulated calculative mindset within a writing task adapted from Reynolds et al. (2021). Participants were randomly assigned to experimental and control groups and completed a brief writing task (approximately 100 words). The materials presented to the experimental group are as follows. Participants were presented with the definition of calculative mindset and the examples of the use of calculative mindset in social relationships. Then, they were asked to imagine themselves supporting this calculative tendency and to write a paragraph expressing this support. The control group was also required to complete a writing task of no fewer than 100 words. The materials presented to the control group are as follows. Participants were presented with the introduction to Hangzhou, which is a famous city in China. Then, they were asked to complete a writing task expressing their opinions about Hangzhou.

We used three items to conduct the *manipulation check*. Three items were adapted from the revised calculative mindset scale in Study 1. A sample question is: “Do you tend to calculate the value of yourself and others?.” The three questions manipulation check used a 7‐point Likert scale. Higher scores indicated a stronger calculative mindset. The Cronbach's *α* coefficient for the three questions was 0.90.

The immoral help scenario was used to measure *ethical compromise*. The immoral help scenario was developed by Yu et al. (2024); participants assume that they were the protagonist in the situation and meet a person who offers them immoral help when facing difficulties.

You are participating in a 1‐month company collective training, need to learn many courses, take graduation exams, and your academic performance will determine your career prospects in the company. Recently, the electronic system in the classroom has been damaged and cannot be repaired for a while. Teachers are unable to use the projector and audio system properly during class, which makes it difficult for people in the back row to hear the training content clearly. You have been arranged to sit in the last row and have been unable to understand the training content during this time. You are worried about the exam. After a day of class, you call your friends and complain that you can't hear the training content clearly, fearing that not being able to pass the training will affect your promotion. Coincidentally, colleague Xiaoming passed by and heard your complaint. Xiao Ming is not in your department and is not very familiar with you on a regular basis. When XiaoMing heard about your difficulties, he/she privately told you that he/she could swap your seat with the front row colleague, although this could lead to that colleague encountering similar difficulties. But Xiaoming said he/she is the person in charge of this training, which is not difficult for him/her, and he/she has not suffered any losses.

*Ethical compromise* was measured using five questions (Yu et al. 2024): “1. To what extent are you willing to accept this help,” “2. To what extent do you feel that help is valuable,” “3. To what extent are you happy with this help,” “4. To what extent are you grateful to the helper,” and “5. To what extent are you willing to deepen your relationship with helpers?.” Scores were given on a 7‐point Likert scale, ranging from 1 “*not at all*” to 7 “*very much*.” Exploratory factor analysis showed a single‐factor structure, explaining 79.55% of the total variation, with interitem correlations ranging from 0.69 to 0.79; thus, we added the scores of the five questions together as the score of receiving immoral help, with higher scores indicating stronger ethical compromise. The Cronbach's *α* coefficient for the five questions was 0.94.

*Situational Moral Disengagement* was measured using two questions. Following the situational moral disengagement measurement by Kish‐Gephart et al. (2014), we selected two dimensions from the eight dimensions of moral disengagement mechanisms that best fit the scenario and designed two questions based on them. The two dimensions and their corresponding questions are as follows. Distribution of consequences: “1. It is okay to accept this help because the loss to colleagues in the front row is not much;” moral justification: “2. Xiao Ming actively offered help to me, and I have no problem accepting this help.” The scores of the two questions were significantly positively correlated (*r* = 0.65, *p* < 0.001). We added the scores of the two questions together as the score of moral disengagement. The higher the score, the stronger the tendency of moral disengagement.

*Emotion* was measured using the PANAS scale (Watson et al. 1988). The PANAS scale includes two subscales measuring positive emotions (such as “attentive”) and negative emotions (such as “irritable”), each subscale consisting of 10 items. Scores were given on a 5‐point Likert scale. The Cronbach's *α* coefficients for positive and negative emotion scales in this study were 0.82 and 0.91, respectively.

We selected demographic variables such as sex, age, educational level as control variables, which are same as Study 1.

#### 4.1.3 Design and Procedure

A one‐way, between‐subject design was adopted. The independent variable was calculative mindset, and the dependent variables were participants' acceptance of immoral help and situational moral disengagement. Participants were randomly assigned to two groups: 142 participants in the experimental group (47.7%) and 156 in the control group (52.3%).

All participants first completed a writing task. Participants in the experimental group completed a writing task related to calculative thinking, while participants in the control group completed a writing task related to Hangzhou. Afterwards, all participants were required to complete the manipulation check. After the manipulation task, participants were required to read the ethical compromise scenario and complete the ethical compromise measurement, the situational moral disengagement measurement, and the PANAS scale. Finally, demographic information was collected.

### 4.2 Results and Discussion

The results of manipulation check were as follows. The calculative mindset scores of participants in the experimental group (*M* = 5.26, SD = 1.21) were significantly higher than those in the control group (*M* = 3.69, SD = 1.74; *t*(296) = 8.92, *p* < 0.001, Cohen's *d* = 1.04), indicating that the experimental manipulation was effective.

Descriptive statistical analysis was conducted for different groups regarding the acceptance of immoral help and moral disengagement (see Table 3).

**TABLE 3:** Descriptive analysis result between experiment group and control group in Study 3 (*n* = 298).

Conditions	Acceptance of immoral helps		Moral disengagements	
	*M*	SD	*M*	SD
Experimental group (*n* = 142)	5.02	1.36	3.65	1.44
Control group (*n* = 156)	4.22	1.70	3.11	1.56

We also conducted the analysis of scores of positive emotion and negative emotion in experimental condition and control condition. Independent samples *t*‐tests showed that the experimental group scored (*M* = 3.46, SD = 0.70) significantly lower than the control group in positive emotion (*M* = 3.65, SD = 0.67; *t*(296) = −2.38, *p* < 0.05, Cohen's *d* = −0.28), while there was no significant difference in negative emotion between the experimental group (*M* = 1.90, SD = 0.80) and the control group (*M* = 1.82, SD = 0.72; *t*(296) = 0.85, *p* = 0.40, Cohen's *d* = 0.01).

Controlling positive emotions, negative emotions, and demographic variables, independent samples *t*‐tests showed that the experimental group scored significantly higher than the control group in situational moral disengagement (*t*(296) = 3.09, *p* < 0.01, Cohen's *d* = 0.36) and acceptance of immoral help (*t*(296) = 4.48, *p* < 0.01, Cohen's *d* = 0.52).

**FIGURE 5:**
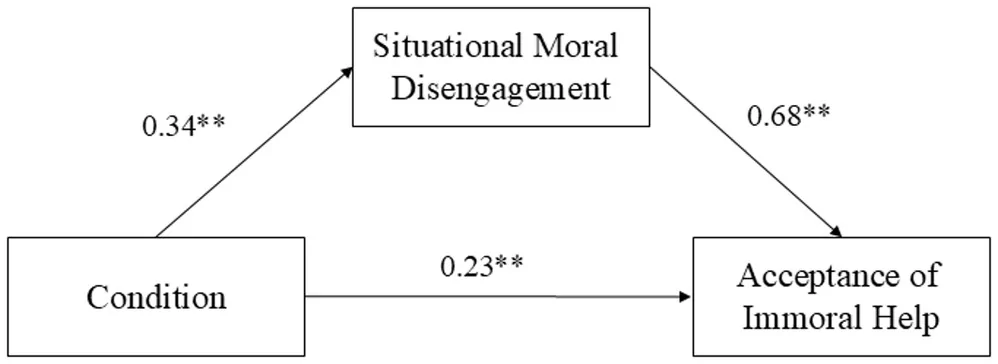
The mediation model of moral disengagement in the relationship between condition and acceptance of immoral help. *Note:* Condition: 0 = “Control group”, 1 = “Experimental group”.

We conducted a mediation analysis (PROCESS v4.1 for SPSS, 5000 bootstraps) with condition as the independent variable, moral disengagement as the mediator and acceptance of immoral help as the dependent variable, controlling for sex, age, educational level, positive emotion and negative emotion. The total effects of condition on acceptance of immoral help (*b* = 0.74, 95% CI [0.38, 1.08], SE = 0.18, *p* < 0.001, b* = 0.46) were significant. Participants in the experimental condition had a stronger tendency for moral disengagement than those in the control condition (*b* = 0.52, 95% CI [0.18, 0.87], SE = 0.18, *p* < 0.01, b* = 0.34). Moral disengagement predicted acceptance of immoral help (*b* = 0.71, 95% CI [0.52, 0.74], SE = 0.04, *p* < 0.001, b* = 0.68). The indirect effect size of moral disengagement was 0.38 (b* = 0.24), with a 95% confidence interval of [0.13, 0.63] that does not include 0, indicating that the mediating effect of situational moral disengagement was significant (Figure 5). This suggests that activating an individual's calculative mindset increases moral disengagement, thereby leading to ethical compromise.

We also examined positive affect and negative affect as alternative mediators. Using experimental condition as the independent variable and willingness to accept immoral help as the dependent variable, we conducted separate mediation analyses for positive affect and negative affect while controlling for gender, age, and education. The indirect effect of positive affect was not significant (*b* = 0.05, 95% CI [−0.06, 0.14], SE = 0.04, b* = 0.03), and the indirect effect of negative affect was also not significant (*b* = 0.03, 95% CI [−0.03, 0.11], SE = 0.04, b* = 0.02). These results further suggest that neither positive affect nor negative affect served as an alternative mechanism underlying the effect of the experimental condition on willingness to accept immoral help in Study 3.

To address alternative causal possibilities, we also tested competing mediation models in which willingness to accept immoral help served as the mediator between condition and moral disengagement. The indirect effect of willingness to accept immoral help in the relationship of condition and moral disengagement was significant. Detailed analyses of the alternative mechanisms can be found in the Supporting Information.

It is also important to note several limitations regarding the manipulation design in Study 3. Specifically, the calculative‐mindset writing task and the control task of writing about Hangzhou may have differed not only in calculative content but also in cognitive load and value orientation. As a result, the observed effect should be interpreted with some caution, as it may partly reflect differences in task demands or value salience across conditions. In addition, although the manipulation check indicated a substantial difference between conditions, it does not fully establish that the manipulation exclusively altered calculative cognition. It remains possible that the writing task also induced a broader rationalization‐oriented attitude that overlaps with, but is not identical to, calculative mindset. Therefore, the findings of Study 3 should be interpreted as providing initial evidence for the effect of a more calculative orientation, rather than as definitively isolating calculative cognition per se. Future research could employ a more closely matched control condition and more fine‐grained manipulation checks to better distinguish calculative cognition from related attitudinal processes.

## 5 General Discussion

The purpose of the persent research is to explore the relationship between the calculative mindset and ethical compromise, as well as the underlying mechanisms. Across two questionnaire studies (Studies 1 and Study 2), calculative mindset was positively associated with higher moral disengagement, which in turn was associated with greater ethical compromise in the economic and status domains. Based on one experimental study (Study 3), calculative mindset led individuals to engage in moral disengagement, which in turn increased their willingness to accept immoral help. This supports previous research findings that calculative mindset triggers higher levels of self‐interested behavior in game tasks (Zhong 2011; Wang et al. 2014). Moreover, this study further suggests that the self‐interest associated with calculative mindset is linked to a greater tendency for individuals to prioritize economic or status gains over moral values, which is in turn associated with ethical compromise. Given that once people make ethical compromises, they may engage in unethical behavior, this study provides a mechanistic explanation for the relationship between calculative mindset and unethical behavior. In addition, this study also supports the proposition that calculative mindset influences moral judgment. Research has found that calculative mindset can lead people to make utilitarian decisions (Wang et al. 2014). In the trolley dilemma, when an individual makes a utilitarian decision of sacrificing one person to save five people, it involves valuing and calculating the number of lives saved, which is also the result of calculative mindset.

The study also supports relational model theory (Rai and Fiske 2011). Relational model theory categorizes interpersonal relationships into communal sharing relationships, authority ranking relationships, equality matching relationships, and market pricing relationships (Gallus et al. 2022). It suggests that individuals in market pricing relationships tend to use proportional thinking to calculate their contributions and others' rewards (Rai and Fiske 2011). Research has found that proportional thinking reduces people's trust in others (Kuzminska et al. 2023) and leads to utilitarian ethical decisions (Zaleskiewicz et al. 2020). Proportional thinking also involves the calculation of social relationships, similar to the calculative mindset. Therefore, the findings of this research support the inference of individual mindset in market pricing relationships. What is worth further exploring is whether individuals with a high calculative mindset tend to characterize their relationships with others as market pricing relationships, which determines socializing strategies and ultimately affects the quality and continuity of interpersonal relationships.

The theoretical contributions of the present research are as follows. First, our findings suggest that individuals with a stronger calculative mindset may show greater willingness to make ethical compromises. This finding deepens our understanding of the moral decision‐making process. Existing studies on moral psychology tend to divide moral decision‐making principles into deontological and utilitarian models (Xu et al. 2020). Individuals with a high calculative mindset tend to be more willing to quantitatively calculate social values which are not supposed to be calculated (Wang et al. 2014; Kim et al. 2022), which makes them more likely to price human life in moral decision‐making or make utilitarian decisions based on people's social status, societal contributions, and the degree of closeness or distance between them and oneself. Therefore, the present research contributes to our understanding of people's moral judgment from dispositional traits. Secondly, this study identifies the mediating role of moral disengagement in the relationship between calculative mindset and ethical compromise. This finding enriches the exploration of the mechanism of ethical compromise. At present, the discussion of ethical compromise mostly focuses on the qualitative analysis of political science and philosophy, and rarely discusses the influencing factors, underlying mechanisms, and subsequent consequences from the individual, interpersonal, and cultural levels. The mediating role of moral disengagement identified in this study reveals the role of social psychological factors in the process of ethical compromise, providing insights into the underlying mechanisms of ethical compromise.

The present research also makes practical contributions. The results suggest that reducing a calculative mindset may help individuals in organizations avoid decisions that involve ethical compromise. Existing research has also shown that a calculative mindset can cause other adverse effects in organizations, such as weakening reciprocal behaviors in organizational contexts (Belmi and Pfeffer 2015) and leading to the objectification of others (Belmi and Schroeder 2021). In interpersonal relationships, we should also reduce the use of a calculative mindset, minimizing the calculation of our own efforts for others and the returns we expect from them. The present research suggests that a calculative mindset may make people more willing to accept help or make decisions that involve ethical compromises, which may create relational costs and undermine the quality of social relationships. Existing research has shown that calculating whether efforts and rewards are proportional can also reduce interpersonal trust in others (Kuzminska et al. 2023). Adopting an exchange‐oriented market mindset toward others can also decrease social mindfulness for others (Xiao and Xin 2024). This suggests that in order to build high‐quality social relationships with others, we may need to reduce our calculative mindset.

However, the present research has some limitations. One limitation is that the participants did not need to bear the consequences of their choice, so it is difficult to objectively evaluate their ethical compromise tendency. In addition, the reliance on self‐report measures may have introduced biases such as social desirability and common method variance. Studies 1 and 2 were based primarily on scenario‐based judgments and attitudinal ratings, and although Study 3 used an experimental design, the dependent variable was still participants' self‐reported willingness to accept immoral help rather than an actual behavioral measure. Therefore, some of the observed associations may have been inflated by self‐report‐related biases, and reported willingness may not fully reflect real‐life behavior. What's more, the causal ordering among calculative mindset, moral disengagement, and ethical compromise remains another important limitation. Although our hypothesized mediation model was theoretically grounded, the cross‐sectional designs of Studies 1 and 2 cannot rule out alternative causal possibilities. For example, ethical compromise may retrospectively trigger moral disengagement as a post hoc justification process. Moreover, omitted dispositional variables, such as empathy, agreeableness, and moral identity, may simultaneously influence these constructs. Future research should use longitudinal, cross‐lagged, or experimental designs and include relevant dispositional controls to clarify the causal ordering among these variables. Another limitation concerns the measurement of ethical compromise in Studies 1 and 2. Although the two items were averaged as an overall evaluative index, they captured related but distinguishable judgments, and item‐level analyses showed that the mediation pattern was more consistent for behavioral acceptability than for moral character evaluation; future research should develop a more comprehensive measure that distinguishes these dimensions. Relatedly, although moral disengagement and ethical compromise were conceptualized as theoretically distinct constructs, the relatively strong association between them in Study 2 suggests that some degree of conceptual or measurement overlap cannot be ruled out. Additional discriminant validity analyses provided only limited evidence for their empirical distinctiveness. Future research should further distinguish these constructs using improved measures, behavioral indicators, time‐lagged designs, or multi‐method assessments. Moreover, all participants were recruited from the Credamo platform and were relatively young, highly educated, and predominantly Chinese. This may limit the generalizability of the findings to broader populations, other cultural contexts, and organizational settings. Future research should test these effects in more diverse samples.

The future directions of this research are as follows. Firstly, we can explore the relationship between calculative mindset and different types of moral decision‐making. Ethical compromise reflects people's choices when secular values such as economic/status gains conflict with moral values. There are also moral dilemmas such as pro‐social lies and unethical pro‐organizational behavior when two or more moral values conflict (Levine and Lupoli 2022; Mishra et al. 2022). It is worth exploring how a calculative mindset influences individuals' responses in these moral dilemmas. Secondly, other underlying mechanisms between calculative mindset and ethical compromise should be explored. The present research verified the mediating role of moral disengagement from the perspective of “calculation.” Previous studies have shown that calculative mindset reduces empathy and the impact of emotion on unethical behavior. Future studies could explore whether this effect/phenomenon might lead to ethical compromises. Thirdly, it is important to explore potential factors that inhibit individuals with calculative mindset from making ethical compromises. Considering the negative consequences of ethical compromise on interpersonal relationships and social adaptation, future studies should investigate whether activating individuals' moral self‐identity, increasing moral sensitivity, or enhancing self‐control can reduce ethical compromise.

## 6 Conclusion

The study concludes with the following findings: a calculative mindset was associated with higher levels of moral disengagement, which in turn was linked to greater willingness to make ethical compromises.

## Funding

This work was supported by the Ministry of Education Humanities and Social Science Project (19YJA190007).

## Ethics Statement

This study was approved by the Medical Ethics Committee of the Department of Psychology and Behavioral Sciences, Zhejiang University. Informed consent was obtained from all participants included in the study. The study was performed in accordance with the ethical standards as laid down in the 1964 Declaration of Helsinki and its later amendments or comparable ethical standards.

## Conflicts of Interest

The authors declare no conflicts of interest.

## Supporting information


**Figure S1:** The mediation model of calculative mindset in the relationship moral disengagement and behavioral acceptability.
**Figure S2:** The mediation model of calculative mindset in the relationship moral disengagement and positive moral character evaluation.
**Figure S3:** The mediation model of behavioral acceptability in the relationship calculative mindset and moral disengagement.
**Figure S4:** The mediation model of positive moral character evaluation in the relationship calculative mindset and moral disengagement.
**Figure S5:** The mediation model of calculative mindset in the relationship moral disengagement and behavioral acceptability.
**Figure S6:** The mediation model of calculative mindset in the relationship moral disengagement and positive moral character evaluation.
**Figure S7:** The mediation model of behavioral acceptability in the relationship calculative mindset and moral disengagement.
**Figure S8:** The mediation model of positive moral character evaluation in the relationship calculative mindset and moral disengagement.
**Figure S9:** The mediation model of acceptance of immoral help in the relationship between condition and moral disengagement.
**Table S1:** GVIF results for the regression model predicting behavioral acceptability in Study 2.
**Table S2:** GVIF results for the regression model predicting positive moral character evaluation in Study 2.
**Table S3:** FDR‐corrected correlations among key variables in Study 1.
**Table S4:** FDR‐corrected mediation‐related path coefficients in Study 1.
**Table S5:** Indirect effect in Study 1 (behavioral acceptability as dependent variable).
**Table S6:** Indirect effect in Study 1 (positive moral character evaluation as dependent variable).
**Table S7:** FDR‐corrected correlations among key variables in Study 2.
**Table S8:** FDR‐corrected mediation‐related path coefficients in Study 2.
**Table S9:** Indirect effect in Study 2 (behavioral acceptability as dependent variable).
**Table S10:** Indirect effect in Study 2 (positive moral character evaluation as dependent variable).
**Table S11:** FDR‐corrected mediation‐related path coefficients in Study 3.
**Table S12:** Indirect effect in Study 3.

## Data Availability

The data that support the findings of this study are available from the corresponding author upon reasonable request.
