# Supplementary material for: Why Prioritize Personal Gain Over Principles? The Relationship Between Calculative Mindset, Moral Disengagement, and Ethical Compromise
Source: Psych J. 2026 Sep 9;15(5):e70120. doi: 10.1002/pchj.70120 (PMC13554994; doi:10.1002/pchj.70120)
Supplement: Supplementary file 1 [file PCHJ-15-e70120-s001.pdf]

The alternative mechanism analyses for each study

Study 1

(1) MD->CM-> Behavioral Acceptability: We conducted a mediation analysis (PROCESS v4.1 for SPSS, 5,000 bootstraps) with moral disengagement as the independent variable, calculative mindset as the mediator and behavioral acceptability as the dependent variable, controlling sex, age and educational level. The total effects of moral disengagement on behavioral acceptability ($b = 0.41$, 95% CI [0.23, 0.60], $SE = .09$, $p < .001$, $b^* = .34$) were significant. Participants higher in moral disengagement reported higher levels of calculative mindset ($b = 0.77$, 95% CI [0.61, 0.93], $SE = .08$, $p < .001$, $b^* = .62$). Calculative mindset was positively associated with behavioral acceptability ($b = 0.20$, 95% CI [0.01, 0.39], $SE = .10$, $p < .05$, $b^* = .21$). The indirect effect via calculative mindset was 0.16 ($b^* = .13$), with a 95% CI [0.02, 0.30] that did not include zero, indicating a significant indirect effect (Figure S1).

Figure S1

The mediation model of calculative mindset in the relationship moral disengagement and behavioral acceptability

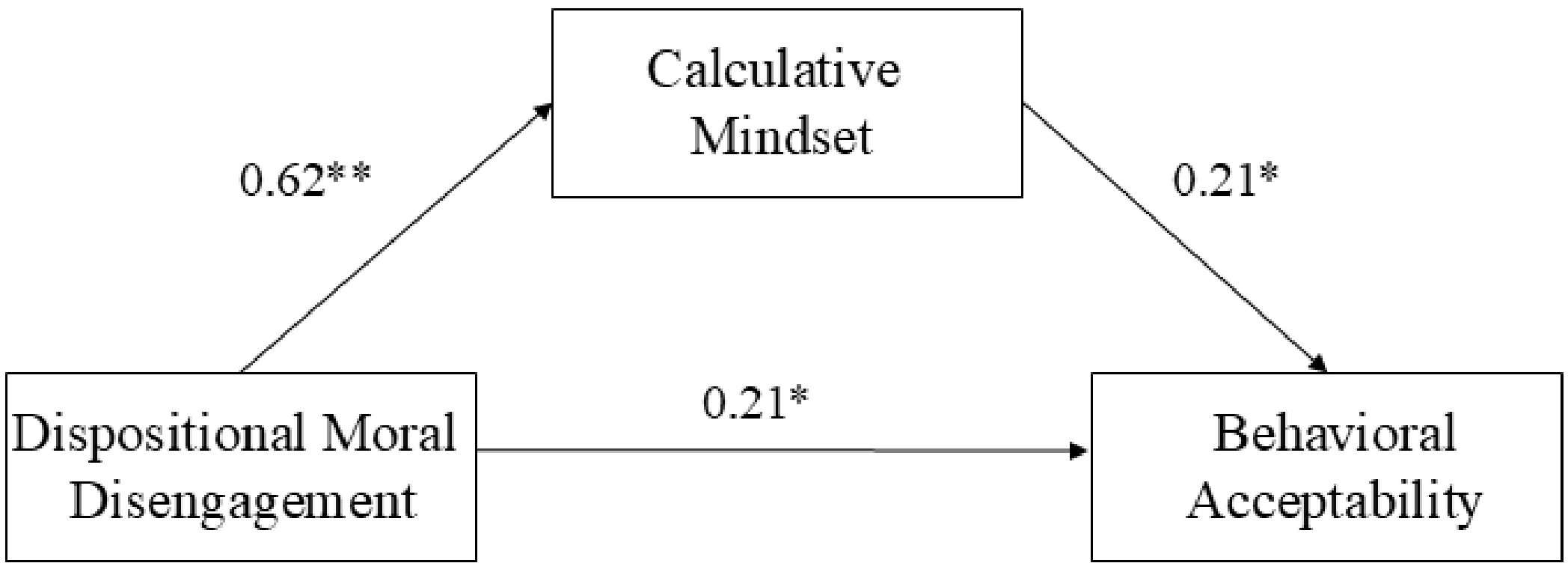

(2) MD->CM-> Positive Moral Character Evaluation: We conducted a mediation analysis (PROCESS v4.1 for SPSS, 5,000 bootstraps) with moral disengagement as the independent variable, calculative mindset as the mediator and positive moral character evaluation as the dependent variable, controlling sex, age and educational level. The total effects of moral disengagement on positive moral character evaluation (b = 0.20, 95% CI [0.06, 0.35], SE = .07, p < .01, b* =.23) were significant. Participants higher in moral disengagement reported higher levels of calculative mindset (b = 0.77, 95% CI [0.61, 0.93], SE = .08, p < .001, b* = .62). The relationship between calculative mindset and positive moral character evaluation was not significant. (b = 0.10, 95% CI [-0.05, 0.25], SE = .08, p = .19, b* = .14). The indirect effect via calculative mindset was 0.08 (b* = .08), with a 95% CI [-0.04, 0.21] including zero, indicating that indirect effect was not significant (Figure S2).

Figure S2

The mediation model of calculative mindset in the relationship moral disengagement and positive moral character evaluation

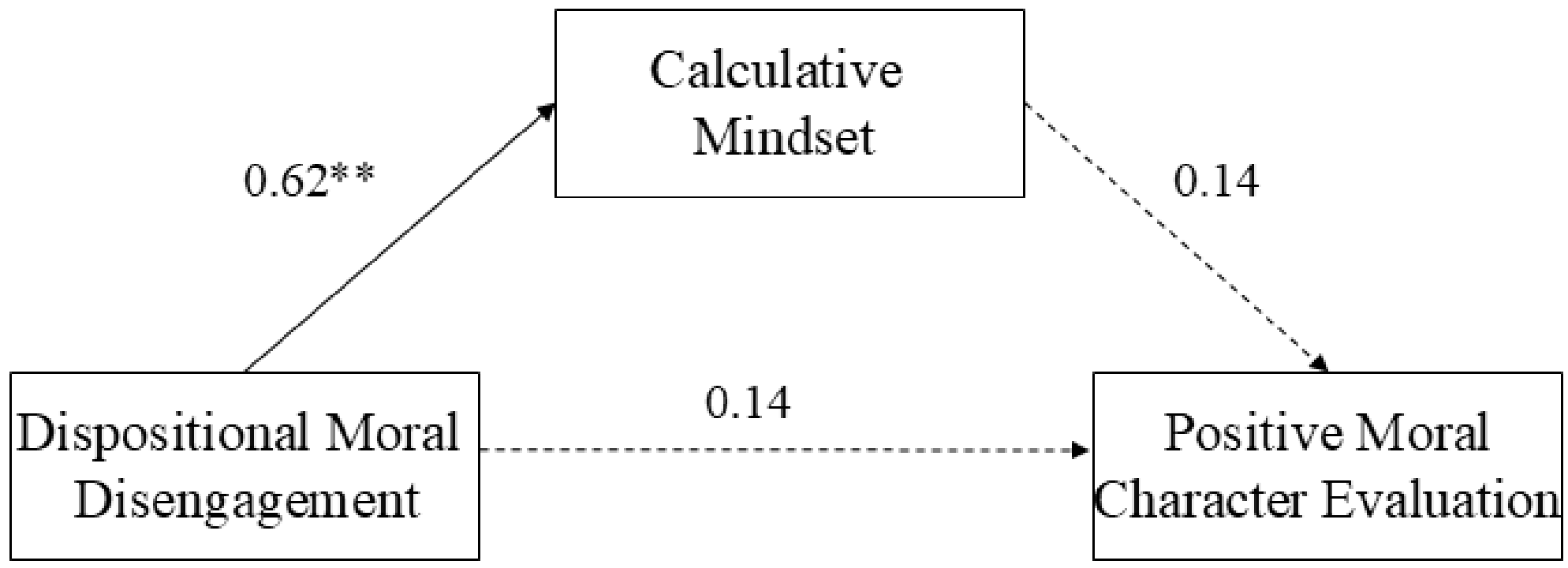

(3) CM->Behavioral Acceptability->MD: We conducted a mediation analysis (PROCESS v4.1 for SPSS, 5,000 bootstraps) with calculative mindset as the independent variable, behavioral acceptability as the mediator and moral disengagement as the dependent variable, controlling sex, age and educational level. The total effects of calculative mindset on moral disengagement (b = 0.50, 95% CI [0.40, 0.61], SE = .05, p < .001, b* = .62) were significant. Participants higher in calculative mindset reported higher levels of behavioral acceptability (b = 0.33, 95% CI [0.18, 0.48], SE = .08, p < .001, b* = .34). Behavioral acceptability was positively associated with moral disengagement (b = 0.12, 95% CI [0.01, 0.24], SE = .06, p < .05, b* = .15). The indirect effect via behavioral acceptability was 0.04 (b* = .05), with a 95% CI [0.01, 0.08] that did not include zero, indicating a significant indirect effect (Figure S3).

Figure S3

The mediation model of behavioral acceptability in the relationship calculative mindset and moral disengagement

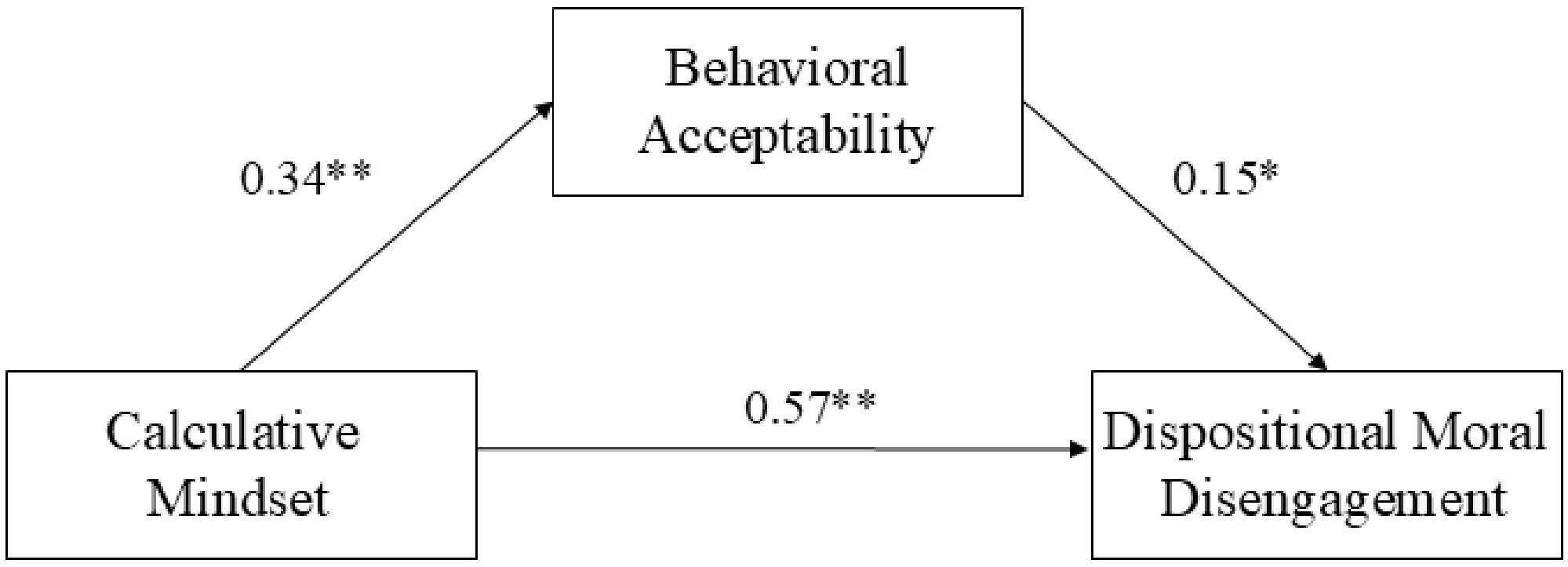

(4) CM-> Positive Moral Character Evaluation ->MD: We conducted a mediation analysis (PROCESS v4.1 for SPSS, 5,000 bootstraps) with calculative mindset as the independent variable, positive moral character evaluation as the mediator and moral disengagement as the dependent variable, controlling sex, age and educational level. The total effects of calculative mindset on moral disengagement (b = 0.50, 95% CI [0.40, 0.61], SE = .05, p < .001, b* = .62) were significant. Participants with a higher calculative mindset tended to evaluate the protagonist's moral character more positively. (b = 0.16, 95% CI [0.05, 0.28], SE = .06, p < .01, b* = .22). The relationship between positive moral character evaluation and moral disengagement was not significant. (b = 0.10, 95% CI [-0.05, 0.25], SE = .08, p = .18, b* = .09). The indirect effect via calculative mindset was 0.02 (b* = .02), with a 95%

CI [-0.01, 0.04] including zero, indicating that indirect effect was not significant (Figure S4).

Figure S4

The mediation model of positive moral character evaluation in the relationship calculative mindset and moral disengagement

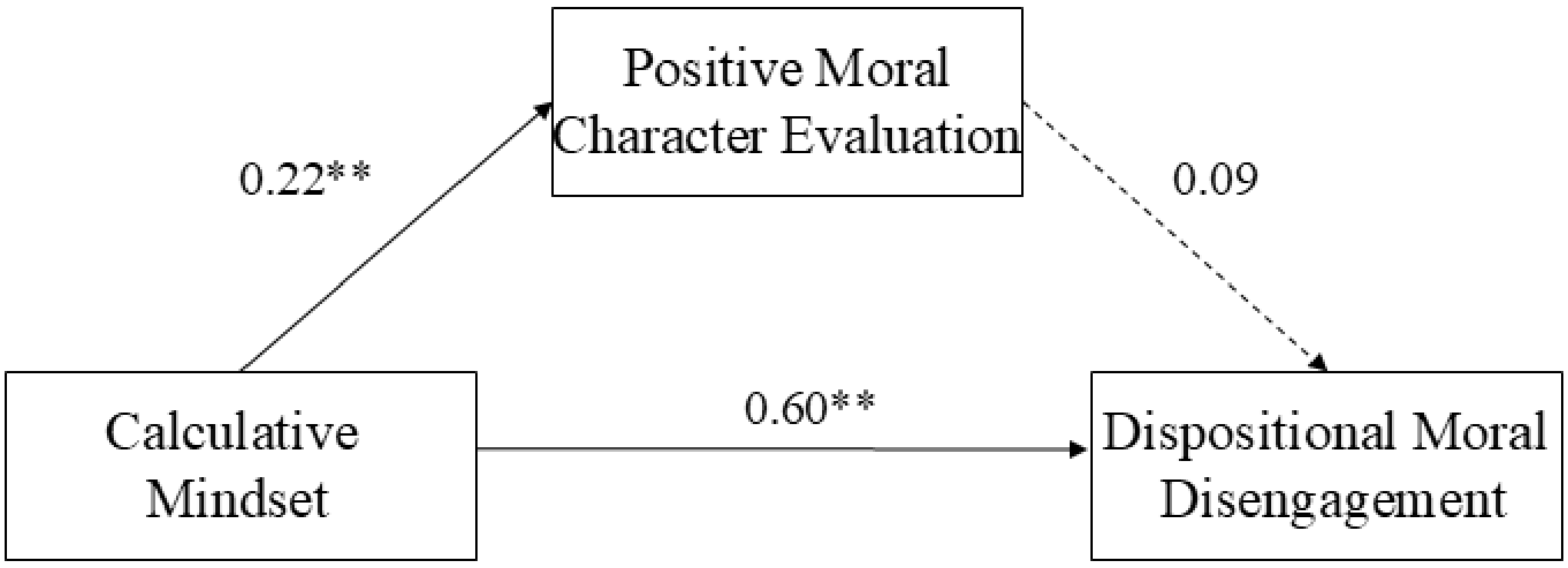

Study 2

(1) MD->CM->Behavioral Acceptability: We conducted a mediation analysis (PROCESS v4.1 for SPSS, 5,000 bootstraps) with moral disengagement as the independent variable, calculative mindset as the mediator and behavioral acceptability as the dependent variable, controlling sex, age and educational level. The total effects of moral disengagement on behavioral acceptability (b = 0.73, 95% CI [0.60, 0.86], SE = .07, p < .001, b* = .69) were significant. Participants higher in moral disengagement reported higher levels of calculative mindset (b = 0.35, 95% CI [0.17, 0.53], SE = .09, p < .001, b* = .31). The effect of calculative mindset on behavioral acceptability was not significant (b = 0.03, 95% CI [-0.09, 0.16], SE = .06, p = .56, b* = .04). The indirect effect via calculative mindset was 0.01 (b* = .01), with a 95% CI [-0.03, 0.06] that included zero, indicating that indirect effect was not significant (Figure S5).

Figure S5

The mediation model of calculative mindset in the relationship moral disengagement and behavioral acceptability

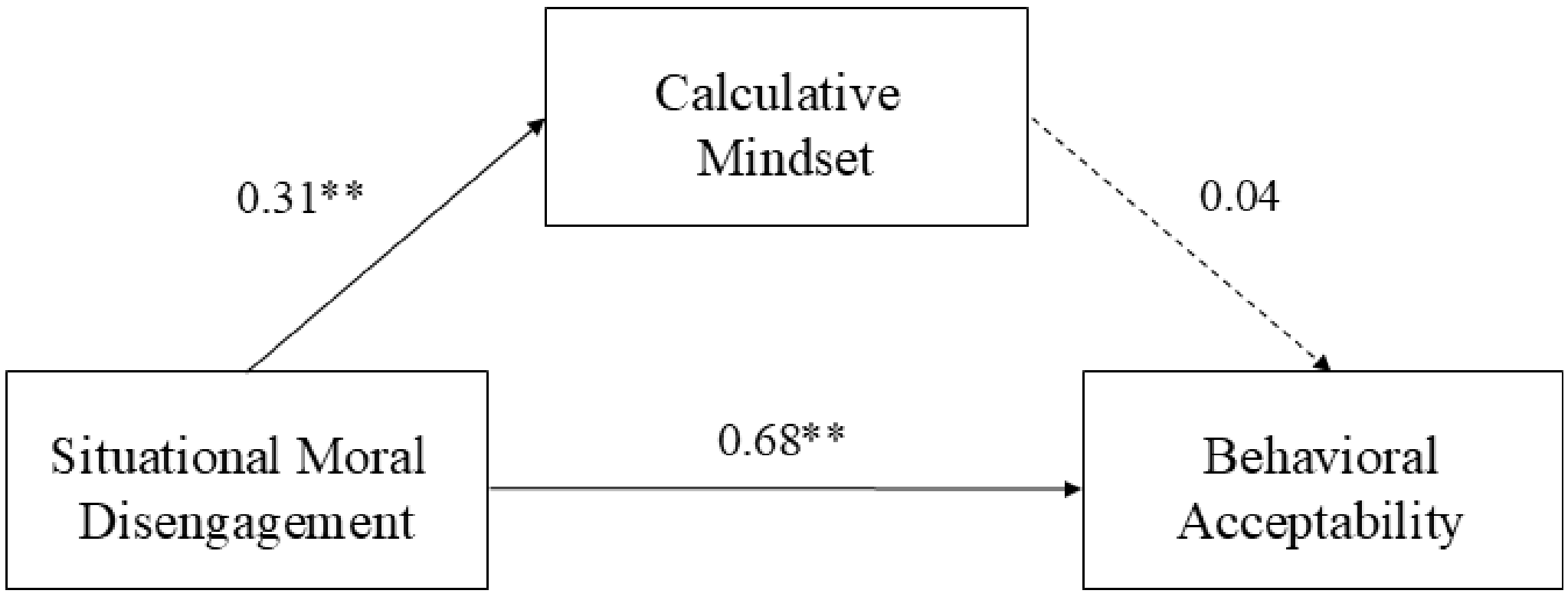

(2) MD->CM-> Positive Moral Character Evaluation: We conducted a mediation analysis (PROCESS v4.1 for SPSS, 5,000 bootstraps) with moral disengagement as the independent variable, calculative mindset as the mediator and positive moral character evaluation as the dependent variable, controlling sex, age and educational level. The total effects of moral disengagement on positive moral character evaluation (b = 0.54, 95% CI [0.42, 0.65], SE = .06, $p < .001$, b* = .64) were significant. Participants higher in moral disengagement reported higher levels of calculative mindset (b = 0.35, 95% CI [0.17, 0.53], SE = .09, $p < .001$, b* = .31). The effect of calculative mindset on positive moral character evaluation was not significant (b = -0.01, 95% CI [-0.12, 0.09], SE = .05, p = .78, b* = -.02). The indirect effect via calculative mindset was -0.005 (b* = -.01), with a 95% CI [-0.05, 0.03] that included zero, indicating that indirect effect was not significant (Figure S6).

Figure S6

The mediation model of calculative mindset in the relationship moral disengagement and positive moral character evaluation

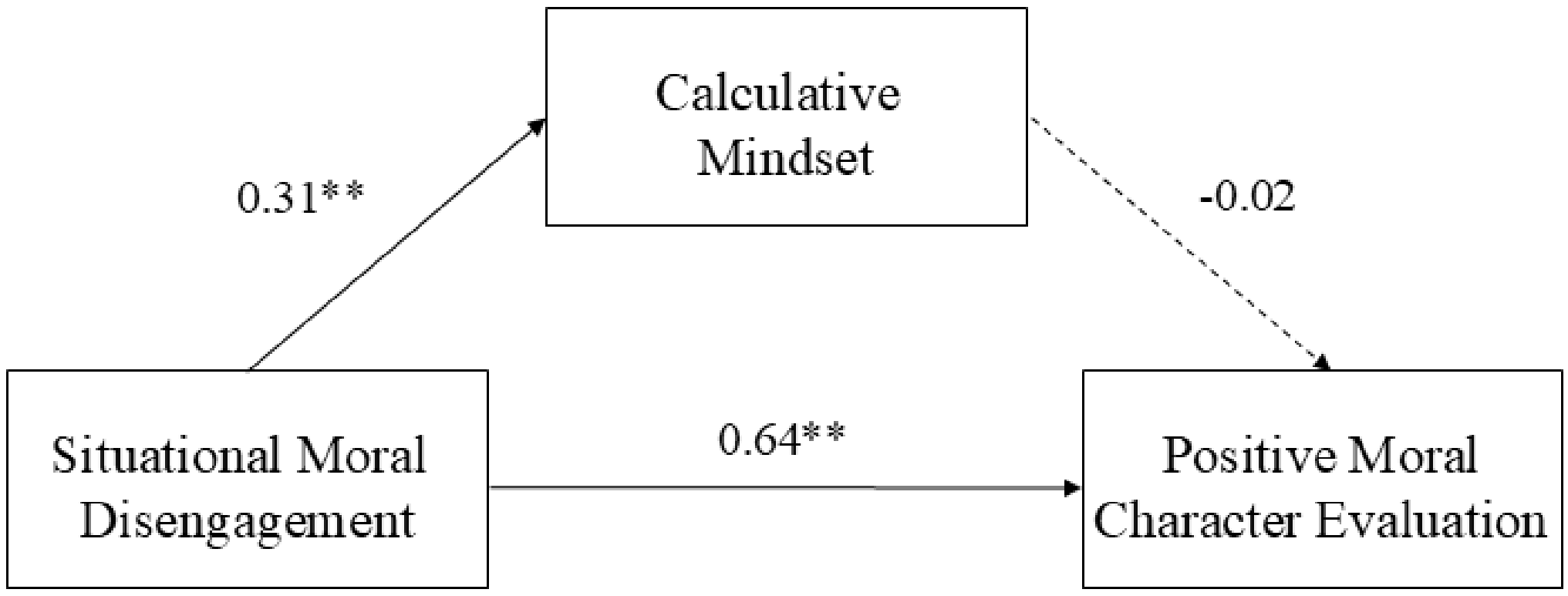

(3) CM->Behavioral Acceptability->MD: We conducted a mediation analysis (PROCESS v4.1 for SPSS, 5,000 bootstraps) with calculative mindset as the independent variable, behavioral acceptability as the mediator and moral disengagement as the dependent variable, controlling sex, age and educational level. The total effects of calculative mindset on moral disengagement ($b = 0.28$, 95% CI [0.13, 0.42], $SE = .07$, $p < .001$, $b^* = .32$) were significant. Participants higher in calculative mindset reported higher levels of behavioral acceptability ($b = 0.24$, 95% CI [0.08, 0.39], $SE = .06$, $p < .01$, $b^* = .26$). Behavioral acceptability was positively associated with moral disengagement ($b = 0.60$, 95% CI [0.49, 0.72], $SE = .06$, $p < .001$, $b^* = .64$). The indirect effect via behavioral acceptability was 0.14 ($b^* = .16$), with a 95% CI [0.04, 0.24] that did not include zero, indicating a significant indirect effect (Figure S7).

Figure S7

The mediation model of behavioral acceptability in the relationship calculative mindset and moral disengagement

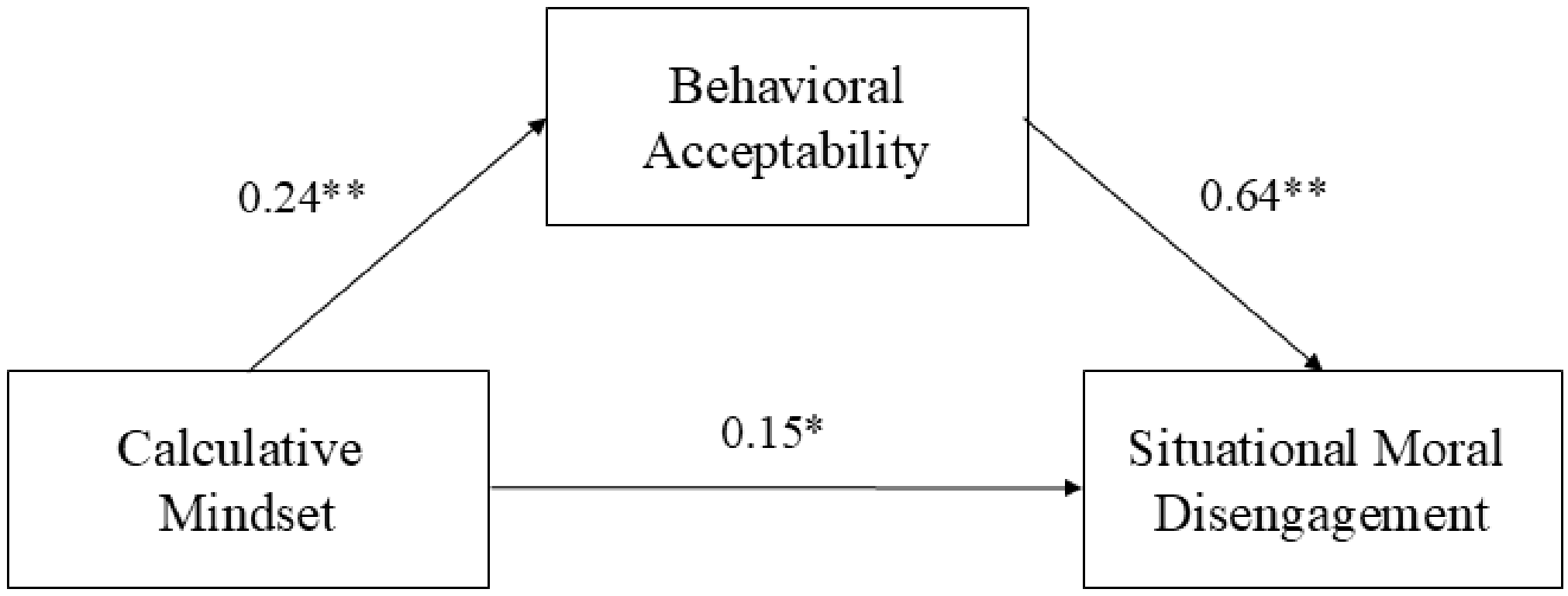

(4) CM-> Positive Moral Character Evaluation ->MD: We conducted a mediation analysis (PROCESS v4.1 for SPSS, 5,000 bootstraps) with calculative mindset as the independent variable, positive moral character evaluation as the mediator and moral disengagement as the dependent variable, controlling sex, age and educational level. The total effects of calculative mindset on moral disengagement (b = 0.28, 95% CI [0.13, 0.42], SE = .07, p < .001, b* = .32) were significant. Participants with a higher calculative mindset tended to evaluate the protagonist's moral character more positively. (b = 0.14, 95% CI [0.01, 0.26], SE = .06, p < .01, b* = .18). Positive moral character evaluation was positively associated with moral disengagement (b = 0.70, 95% CI [0.54, 0.85], SE = .08, p < .001, b* = .58). The indirect effect via positive moral character evaluation was 0.09 (b* = .11), with a 95%

CI [0.003, 0.19] that did not include zero, indicating a significant indirect effect (Figure S8).

Figure S8

The mediation model of positive moral character evaluation in the relationship calculative mindset and moral disengagement

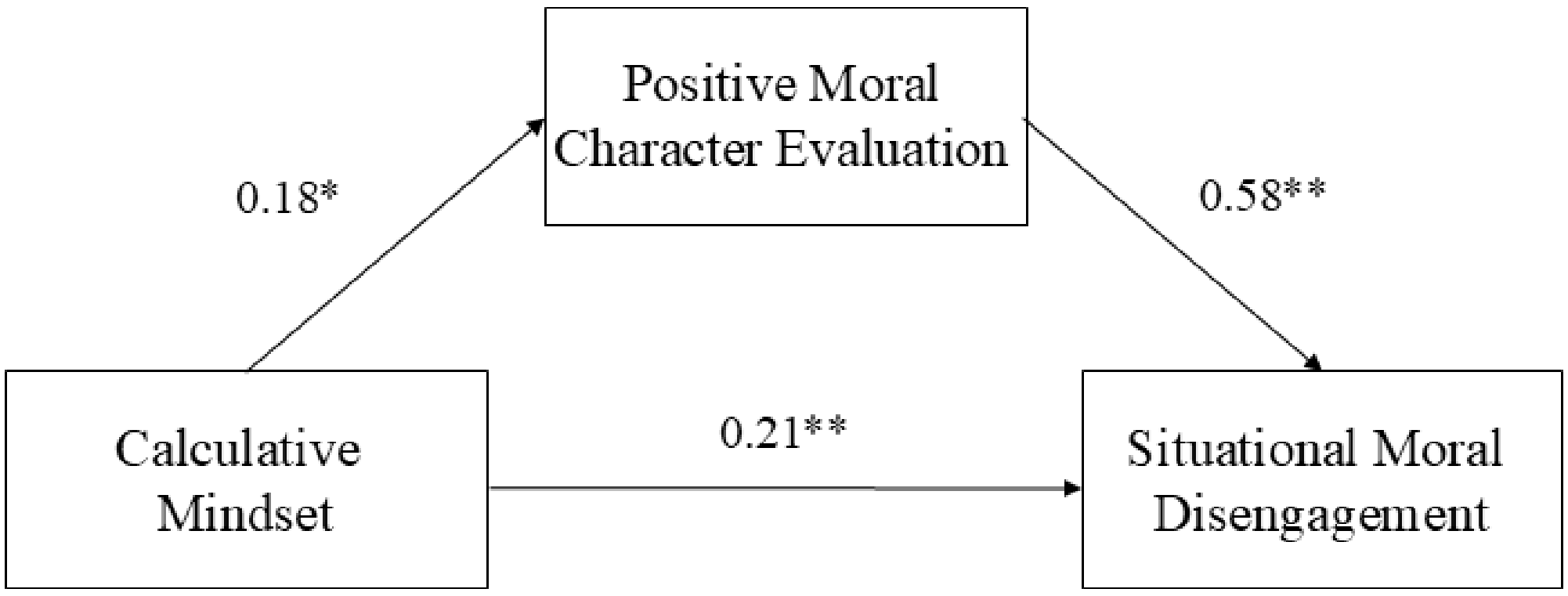

Study 3

Condition->EC->MD We conducted a mediation analysis (PROCESS v4.1 for SPSS, 5,000 bootstraps) with condition as the independent variable, moral disengagement as the mediator and acceptance of immoral help as the dependent variable, controlling sex, age, educational level, positive emotion and negative emotion. The total effects of condition on moral disengagement (b = 0.52, 95% CI [0.18, 0.87], SE = .18, p < .01, $b*$ = .34) were significant. Participants in experimental condition had a stronger tendency of acceptance of immoral help than control condition (b = 0.74, 95% CI [0.38, 1.08], SE = .18, p < .001, $b*$ = .46). Acceptance of immoral help predicted moral disengagement (b = 0.70, 95% CI [0.62, 0.78], SE = .04, p < .001, b* = .74). The indirect effect size of moral disengagement was 0.52 (b* = .34), with a 95% confidence interval of [0.27, 0.77] that does not include 0, indicating that the mediating effects of situational moral disengagement was significant (see Figure S9).

Figure S9

The mediation model of acceptance of immoral help in the relationship between condition and moral disengagement.

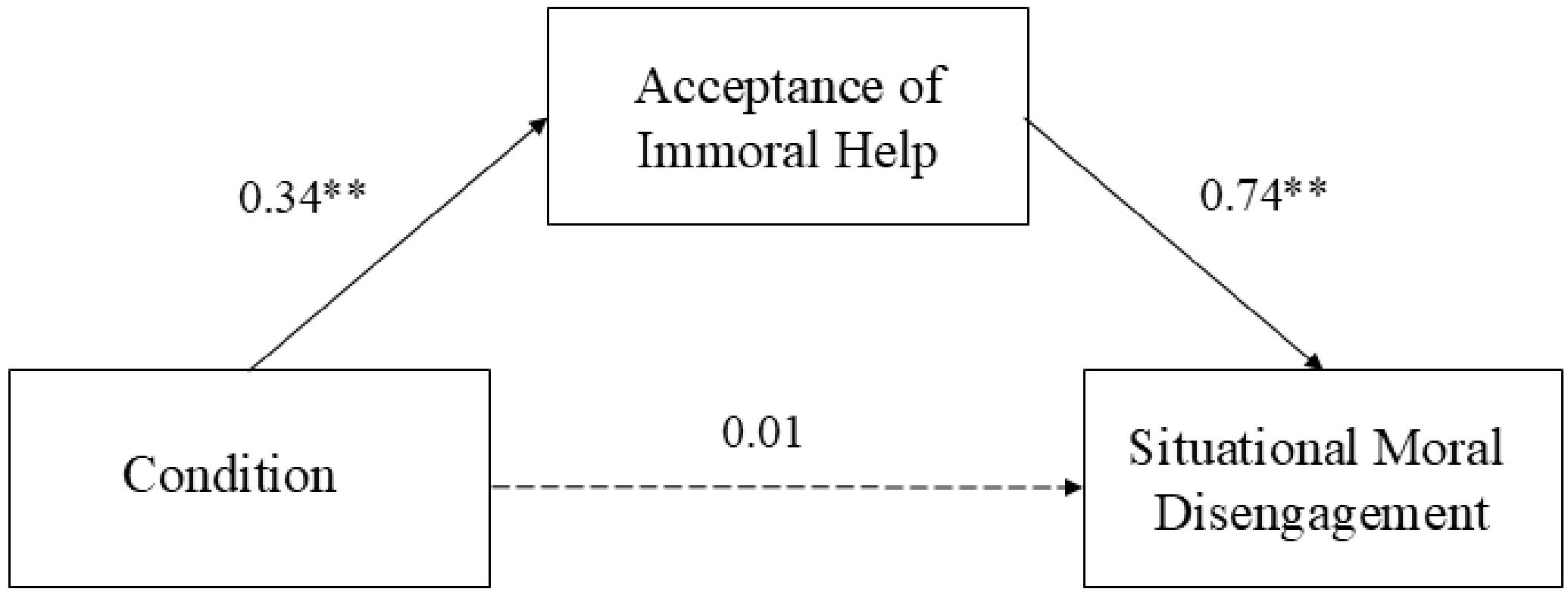

VIF statistics for multicollinearity in study 2

We assessed multicollinearity by calculating variance inflation factors (VIFs) using the car package in R for the regression model predicting behavioral acceptability and positive moral character evaluation, respectively. In both models, calculative mindset, moral disengagement, gender, age, and education were included as predictors. Because some control variables were categorical, generalized variance inflation factors (GVIFs) were obtained, and the adjusted values, GVIF^(1/(2×df)), were interpreted. As shown in Table S1 and S2, the adjusted VIF values were identical across the two outcome models and ranged from 1.021 to 1.111, well below the commonly used cutoff of 5 and the more conservative cutoff of 3. These results suggest that multicollinearity was not a serious concern.

Table S1

GVIF results for the regression model predicting behavioral acceptability in Study 2

**Predictor**	**GVIF**	**df**	**Adjusted VIF, GVIF^(1/(2×df))**
Calculative mindset	1.234	1	1.111
Moral disengagement	1.152	1	1.073
Gender	1.042	1	1.021
Age	1.189	4	1.022
Education	1.110	2	1.026

Table S2

GVIF results for the regression model predicting positive moral character evaluation in Study 2

**Predictor**	**GVIF**	**df**	**Adjusted VIF, GVIF^(1/(2×df))**
Calculative mindset	1.234	1	1.111
Moral disengagement	1.152	1	1.073
Gender	1.042	1	1.021
Age	1.189	4	1.022
Education	1.110	2	1.026

Multiple-Testing Correction in each study

Study 1

To address the issue of multiple testing, we applied the Benjamini–Hochberg false discovery rate correction to the relevant p-values in Study 1. Specifically, FDR correction was applied to the correlation analyses among calculative mindset, moral disengagement, and ethical compromise, as well as to the mediation-related path coefficients. The mediation model included sex, age, and educational background as covariates. The indirect effect was estimated using 5,000 bootstrap samples.

FDR-Corrected Correlation Results

As shown in Table S3, the correlations among calculative mindset, moral disengagement, behavioral acceptability and positive moral character evaluation remained significant after FDR correction. Calculative mindset was positively correlated with moral disengagement, behavioral acceptability and positive moral character evaluation. Moral disengagement was also positively correlated with behavioral acceptability and positive moral character evaluation. Behavioral acceptability was positively correlated with positive moral character evaluation.

Table S3

FDR-Corrected Correlations Among Key Variables in Study 1

Analysis	r	Original p	FDR-adjusted p
Calculative mindset – Moral disengagement	.627	< .001	< .001
Calculative mindset –Behavioral acceptability	.352	< .001	< .001
Calculative mindset – Positive moral character evaluation	.239	<.01	<.01
Moral disengagement – Behavioral acceptability	.365	< .001	< .001
Moral disengagement – Positive moral character evaluation	.247	<.01	<.01
Behavioral acceptability – Positive moral character evaluation	.566	<.001	<.001

FDR-Corrected Mediation-Related Path Coefficients

We then examined whether the mediation-related path coefficients remained significant after FDR correction. As shown in Table S4, calculative mindset significantly predicted moral disengagement after correction. Moral disengagement significantly predicted behavioral acceptability after correction, while moral

disengagement didn't significantly predict positive moral character evaluation after correction. The direct effect of calculative mindset on behavioral acceptability remained significant after correction, although this effect was close to the conventional significance threshold. However, the direct effect of calculative mindset on positive moral character evaluation remained insignificant after correction.

Table S4

FDR-Corrected Mediation-Related Path Coefficients in Study 1

Path	b	SE	z	b*	Original p	FDR-adjusted p
Calculative mindset → Moral disengagement	0.504	0.058	8.741	.622	< .001	< .001
Moral disengagement → Behavioral acceptability	0.254	0.104	2.453	.211	.014	.035
Calculative mindset → Behavioral acceptability	0.202	0.092	2.196	.207	.028	.047
Moral disengagement → Positive moral character evaluation	0.128	0.090	1.413	.141	.158	.197
Calculative mindset →Positive moral character evaluation	0.100	0.081	1.235	0.137	0.217	0.217

Indirect Effect

As shown in Table S5, S6, the indirect effect of calculative mindset on behavioral acceptability through moral disengagement remained significant. The 95% bootstrap confidence interval did not include zero, supporting the hypothesized indirect effect after controlling for sex, age, and educational level. The indirect effect of calculative mindset on positive moral character evaluation through moral disengagement remained insignificant after controlling for sex, age, and educational level.

Table S5

Indirect Effect in Study 1 (Behavioral acceptability as dependent variable)

Effect	b	SE	z	b*	Original p	FDR-adjusted p	95% Bootstrap CI
Indirect effect	0.128	0.056	2.300	.131	.022	.029	[0.027, 0.248]
Total effect	0.330	0.076	4.361	.338	< .001	< .001	[0.187, 0.484]

Table S6

Indirect Effect in Study 1 (Positive moral character evaluation as dependent variable)

Effect	b	SE	z	b*	Original p	FDR-adjusted p	95% Bootstrap CI
Indirect effect	0.064	0.046	1.392	.088	.164	.164	[-0.025, 0.157]
Total effect	0.164	0.061	2.676	.225	.007	.015	[0.043, 0.285]

Study 2

The corrected analysis conducted in Study 2 was the same as that conducted in Study 1.

FDR-Corrected Correlation Results

As shown in Table S7, the correlations among calculative mindset, moral disengagement, behavioral acceptability and positive moral character evaluation remained significant after FDR correction. Calculative mindset was positively correlated with moral disengagement, behavioral acceptability and positive moral character evaluation. Moral disengagement was positively correlated with behavioral acceptability and positive moral character evaluation. Behavioral acceptability was positively correlated with positive moral character evaluation.

Table S7

FDR-Corrected Correlations Among Key Variables in Study 2

Analysis	r	Original p	FDR-adjusted p
Calculative mindset – Moral disengagement	.341	< .001	< .001
Calculative mindset – Behavioral acceptability	.246	.003	.004
Calculative mindset – Positive moral character evaluation	.192	.023	.023
Moral disengagement – Behavioral acceptability	.676	< .001	< .001
Moral disengagement – Positive moral character evaluation	.631	<.001	<.001
Behavioral acceptability – Positive moral character evaluation	.664	<.001	<.001

FDR-Corrected Mediation-Related Path Coefficients

We then examined whether the mediation-related path coefficients remained significant after FDR correction. As shown in Table S8, calculative mindset significantly predicted moral disengagement after correction. Moral disengagement also significantly predicted behavioral acceptability and positive moral character evaluation after correction. The direct effect of calculative mindset on behavioral acceptability and positive moral character evaluation was not significant after accounting for moral disengagement and covariates.

Table S8

FDR-Corrected Mediation-Related Path Coefficients in Study 2

Path	b	SE	z	b*	Original p	FDR-adjusted p
Calculative mindset → Moral disengagement	0.277	0.076	3.645	.317	< .001	.001
Moral disengagement → Behavioral acceptability	0.715	0.080	8.889	.680	< .001	< .001
Moral disengagement → Positive moral character evaluation	0.541	0.064	8.455	.644	<.001	<.001
Calculative mindset → Behavioral acceptability	0.037	0.064	0.576	.040	.565	.565
Calculative mindset → Positive moral character evaluation	-0.015	0.051	-0.290	-0.020	0.772	0.772

Indirect Effect

As shown in Table S9, S10, the indirect effect of calculative mindset on behavioral acceptability and positive moral character evaluation through moral disengagement remained significant. The 95% bootstrap confidence interval did not include zero, supporting the hypothesized indirect effect after controlling for sex, age, and educational level.

Table S9

Indirect Effect in Study 2 (Behavioral acceptability as dependent variable)

Effect	b	SE	z	b*	Original p	FDR-adjusted p	95% Bootstrap CI
Indirect	0.198	0.060	3.304	.215	<.001	.003	[0.086, 0.327]
Total effect	0.235	0.083	2.823	.255	.005	.006	[0.070, 0.397]

Table S10

Indirect Effect in Study 2 (Positive moral character evaluation as dependent variable)

Effect	b	SE	z	b*	Original p	FDR-adjusted p	95% Bootstrap CI
Indirect	0.150	0.047	3.170	.204	.002	.003	[0.064, 0.251]
Total effect	0.135	0.066	2.034	.184	.042	.042	[0.003, 0.268]

Study 3

To address the issue of multiple testing, we applied the Benjamini–Hochberg false discovery rate correction to the relevant p-values in Study 3. Because Study 3 used an experimental design, the experimental condition was dummy-coded before being entered into the mediation model. The model specified experimental condition as the independent variable, moral disengagement as the mediator, and ethical compromise as the dependent variable. Sex, age, educational level, positive emotion, and negative emotion were included as covariates. The indirect effect was estimated using 5,000 bootstrap samples.

FDR-Corrected Mediation-Related Path Coefficients

As shown in Table S11, the core mediation-related paths remained significant after FDR correction. Experimental condition significantly predicted moral disengagement, and moral disengagement significantly predicted ethical compromise after correction.

Table S11

FDR-Corrected Mediation-Related Path Coefficients in Study 3

Path	b	SE	z	b*	Original p	FDR-adjusted p
Experimental condition → Moral disengagement	0.526	0.175	3.010	.173	.003	.003
Moral disengagement → Ethical compromise	0.713	0.040	17.936	.681	< .001	< .001

Indirect Effect

As shown in Table S12, the indirect effect of experimental condition on ethical compromise through moral disengagement remained significant after correction. The 95% bootstrap confidence interval did not include zero, supporting the hypothesized indirect effect after controlling for sex, age, educational level, positive emotion, and negative emotion.

Table S12

Indirect Effect in Study 3

Effect	b	SE	z	b*	Original p	FDR-adjusted p	95% Bootstrap CI
Indirect effect	0.375	0.127	2.960	.118	.003	.003	[0.128, 0.620]
Total effect	0.735	0.177	4.157	.231	< .001	< .001	[0.385, 1.082]
